# Three proliferating cell nuclear antigen homologues from *Metallosphaera sedula* form a head-to-tail heterotrimer

**DOI:** 10.1038/srep26588

**Published:** 2016-05-27

**Authors:** Fumiya Iwata, Hidehiko Hirakawa, Teruyuki Nagamune

**Affiliations:** 1 Department of Chemistry and Biotechnology, School of Engineering, The University of Tokyo, 7-3-1 Hongo, Bunkyo-ku, Tokyo 113-8656, Japan; 2 Department of Bioengineering, School of Engineering, The University of Tokyo, 7-3-1 Hongo, Bunkyo-ku, Tokyo 113-8656, Japan

## Abstract

Proliferating cell nuclear antigen (PCNA) is a sliding clamp that plays a key role in DNA metabolism. Genome sequence analysis has revealed that some crenarchaea possess three *PCNA* genes in their genome, but it has been reported that three PCNAs do not always form a unique heterotrimer composed of one of each molecule. The thermoacidophilic archaeon, *Metallosphaera sedula*, has three *PCNA* homologue genes. Here, we demonstrated that the three PCNA homologues, MsePCNA1, MsePCNA2 and MsePCNA3, exclusively form a heterotrimer in a stepwise fashion; MsePCNA1 and MsePCNA2 form a heterodimer, and then MsePCNA3 binds to the heterodimer. We determined that the dissociation constants between MsePCNA1 and MsePCNA2, and between MsePCNA3 and the MsePCNA1:MsePCNA2 heterodimer are 0.29 and 43 nM, respectively. Moreover, the MsePCNA1, MsePCNA2 and MsePCNA3 heterotrimer stimulated *M. sedula* DNA ligase 1 activity, suggesting that the heterotrimer works as a DNA sliding clamp in the organism. The stable and stepwise heterotrimerization of *M. sedula* PCNA homologues would be useful to generate functional protein-based materials such as artificial multi-enzyme complexes, functional hydrogels and protein fibres, which have recently been achieved by protein self-assembly.

DNA metabolism such as DNA replication and repair is a vital process for all organisms. In such processes, DNA sliding clamp, which is a ring-shaped protein with a central cavity to encircle duplex DNA, plays a central role by recruiting DNA related proteins for accurate and efficient DNA replication and repair^1^,^2^,^3^. Bacterial sliding clamps, β-clamps, are dimeric proteins^4^, while eukaryotic ones, proliferating cell nuclear antigens (PCNAs), are trimeric proteins^5^. Eukaryotic PCNA consists of three identical subunits, in which two domains are connected by an interdomain-connecting loop (IDCL). PCNA works as a scaffold to tether DNA metabolizing enzymes to the DNA by binding its C-terminal region and IDCL to a specific binding motif of PCNA-interacting proteins (PCNA interacting protein box, PIP-box)^6^. Replication factor C (clamp loader) forms a complex with PCNA and opens the stable homotrimeric PCNA ring structure (the dissociation constant of human PCNA is ~20 nM^7^) in an ATP-dependent manner to load on duplex DNA^8^. Loaded PCNA tethers DNA polymerases, DNA ligase, endonucleases and topoisomerase to the DNA for efficient and accurate DNA replication^9^. In addition to the above enzymes, glycosylases, mispair binding proteins and helicases are recruited by PCNA for DNA repair^10^. PCNA also interacts with cell cycle proteins, histone chaperones and sister-chromatid cohesion factors, and thereby plays important roles in cell cycle regulation, chromatin assembly and distribution of replicated chromosomes^11^.

Archaea have PCNA as a sliding clamp, though the cellular structure is similar to that of the bacterial one^12^. Although there is low sequence similarity between archaeal and eukaryotic PCNAs, the overall structure and function of archaeal PCNA are similar to those of eukaryotic PCNAs^13^,^14^,^15^,^16^,^17^,^18^. However, several unique features have been found in the archaeal PCNAs. *Pyrococcus furiosus* DNA polymerase B forms two kinds of complexes with PCNA because of a secondary interaction in addition to the PIP-Box interaction, and consequently its function is switched from polymerase to exonuclease by the configuration of the PCNA-enzyme complex^19^. The secondary interaction has also been found in a complex of *Archaeoglobus fulgidus* PCNA and RNaseH II, which removes RNA primers from Okazaki fragment junctions and cleaves misincorporated ribonucleotides, and changes the orientation of the enzyme for DNA to access the substrate ribonucleotides^20^.

Eukaryotes have single PCNAs, but some archaea have multiple *PCNA* genes. The euryarchaeon, *Thermococcus kodakaraensis*, and the crenarchaeon, *Pyrobaclum aerophilum*, possess two PCNAs. Two *T. kodakaraensis* PCNAs separately form homotrimers and stimulate DNA polymerase activity, but only one is necessary for the vitality of the organism^21^. One of two PCNA homologues from *P. aerophilum* strongly interacts with flap endonuclease, family 4 uracil DNA glycosylase and DNA polymerase B3, and therefore functions a PCNA, while the other homologue weakly interacts with the enzymes^22^. Interestingly, three PCNAs have been found in the crenarchaeota, *Sulfolobus solfataricus*, *S. tokodaii* and *Aeropyrum pernix*, and are reported to form heterotrimers^23^,^24^,^25^.

*S. solfataricus* PCNA is the first discovered heterotrimeric DNA sliding clamp. The three distinct PCNA subunits are monomeric proteins alone that form a heterotrimer in a stepwise association^17^. Each *S. solfataricus* PCNA subunit interacts with specific DNA metabolising enzymes; SsoPCNA1 interacts with flap endonuclease 1^26^ and Y-family polymerase Dpo4^27^, SsoPCNA2 interacts with DNA polymerase B1^28^, and SsoPCNA3 interacts with DNA ligase 1^29^ and family 4 uracil DNA glycosylase^30^. The assembly of flap endonuclease 1, DNA polymerase B1 and DNA ligase 1 on the PCNA ring has been reported to enhance an Okazaki fragment maturation due to the sequential enzyme cascade reaction mechanism^31^,^32^.

It is necessary to experimentally show the existence of exclusive heterotrimerisation even if three distinct *PCNA* genes are found in a genome. PCNAs from *S. tokodaii* and *A. pernix*, which form heterotimers, also form a heterotetramer^33^ and a homotrimer^25^ *in vitro*, respectively. These findings indicate that the existence of three distinct *PCNA* genes does not always indicate the formation of a unique heterotrimer composed of three distinct subunits.

The thermoacidophilic crenarchaeon, *Metallosphaera sedula*, which grows on elemental sulfur and metal sulphides at 50 to 80 °C and pH 2 to 3, and can mobilise metals from metal sulfides^34^, has three distinct PCNA subunit homologues. In this study, we report that the homologues exclusively formed a head-to-tail heterotrimer in a stepwise manner: first, two homologues formed a heterodimer, and then the other homologue formed a heterotrimer with the heterodimer. Surface plasmon resonance analysis revealed that the *M. sedula* heterotrimer is more stable than the *S. solfataricus* PCNA heterotrimer. Furthermore, the heterotrimer stimulated the nick closing activity of DNA ligase 1, suggesting that the heterotrimer works as a sliding clamp. Recently, artificial protein self-assembly has attracted great interest in biotechnology for developing functional materials such as hydrogels for cell stimulation^35^, nanofibres for multivalent antibody response^36^, and protein complexes for multi-enzymatic reactions^37^,^38^. We have also demonstrated that selective and stepwise heterotrimerisation of *S. solfataricus* PCNA subunits were promising scaffold proteins to generate an artificial multi-enzyme complex^39^ and protein gel encapsulating multiple enzymes^40^. *M. sedula* PCNA homologues that form a stable heterotrimer in a stepwise manner would be useful for constructing functional protein-based materials.

## Results and Discussion

### *M. sedula* PCNA homologues have high sequence identity with *S. solfataricus* PCNAs

A genome survey of crenarchaea revealed that each of the organisms belonging to the orders Desulfurococcales, Sulfolobales, Acidilobales and Fervidicoccales has three distinct PCNA homologue genes, and each of those belonging to the order Thermoproteales has one or two homologue genes (Supplementary Table 1). *S. solfataricus*, which belongs to the order Sulfolobales, has three PCNAs, SsoPCNA1, SsoPCNA2 and SsoPCNA3, which are monomeric proteins that form a ring-shaped heterotrimer in a stepwise manner; SsoPCNA1 and SsoPCNA2 form a stable heterodimer, and then SsoPCNA3 associates with the heterodimer to form a heterotrimer^28^. *A. pernix* belongs to the order Desulfurococcales and has three PCNAs, which form a heterotrimer, but one of which forms a homotrimer^25^. Thus, we speculated that PCNAs from organisms belonging to the order Sulfolobales exclusively form heterotrimers.

**Figure 1:**
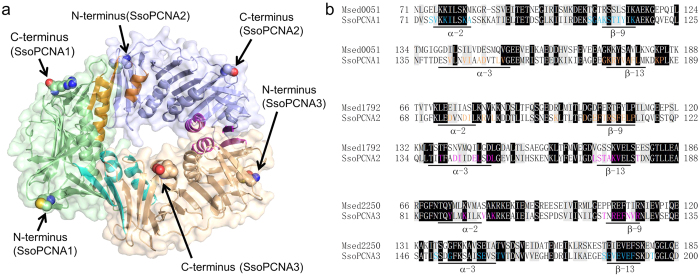
Amino acid residues located on the interfaces between the *S. solfataricus* PCNA subunits. (**a**) Crystal structure of the *S. solfataricus* PCNA heterotrimer. The helices (α-2 and α-3) and strands (β-9 and β-13) at the interfaces between SsoPCNA1 and SsoPCNA2, SsoPCNA2 and SsoPCNA3, and SsoPCNA3 and SsoPCNA1 are shown in orange, magenta and cyan, respectively. The N- and C- termini are shown as spheres. (**b**) Partial sequence alignments between the *M. sedula* PCNA homologues and the *S. solfataricus* PCNAs. Invariant residues are highlighted in black and highly conserved residues are highlighted in grey. Amino acid residues exposed on the interface between SsoPCNA1 and SsoPCNA2, SsoPCNA2 and SsoPCNA3, and SsoPCNA3 and SsoPCNA1 are shown in orange, magenta and cyan, respectively. Whole sequence alignments are shown in Supplementary Fig. S1.

The three PCNA homologues from *M. sedula*, Msed_0051 (UniProt: A4YCS6), Msed_1792 (UniProt: A4YHP5) and Msed_2250 (UniProt: A4YIY6), have the highest identity with SsoPCNA1 (41%), SsoPCNA2 (53%) and SsoPCNA3 (46%), respectively (Supplementary Table 2). *S. solfataricus* PCNA subunits interact with each other in a head-to-tail fashion: the α-2 helices and β-9 strands of SsoPCNA1, SsoPCNA3 and SsoPCNA2 form interfaces with the α-3 helices and β-13 strands of SsoPCNA3, SsoPCNA2 and SsoPCNA1, respectively (Fig. 1a). The residues exposed to the interfaces in the region of these helices and strands are well conserved in the *M. sedula* PCNA homologues (Fig. 1b). This similarity suggests that *M. sedula* PCNA homologues are monomeric proteins that exclusively form a heterotrimer similarly to *S. solfataricus* PCNAs. Thus, Msed_0051, Msed_1792 and Msed_2250 were named MsePCNA1, MsePCNA2 and MsePCNA3, respectively.

### *M. sedula* PCNA homologues form a hetero-complex in a stepwise manner

**Figure 2:**
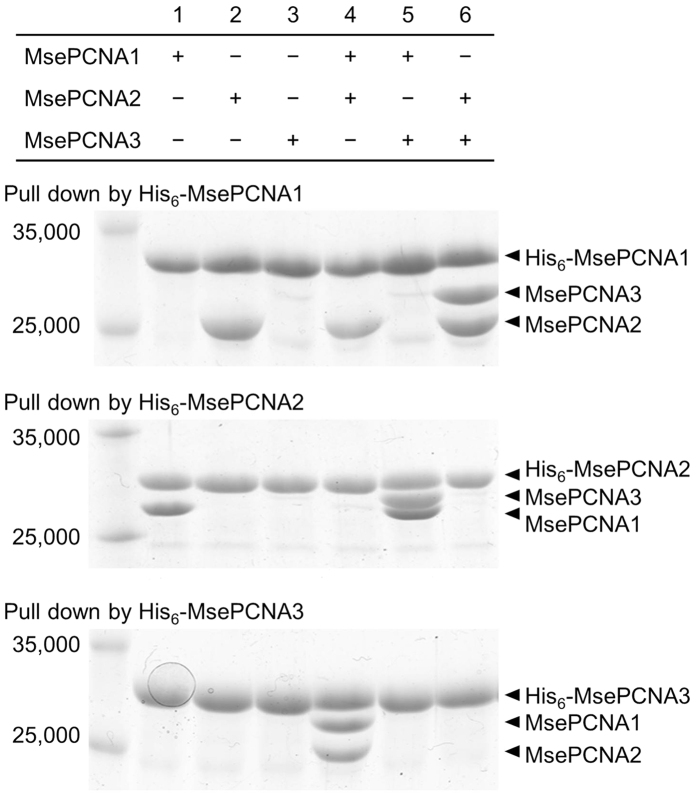
Interactions among the *M. sedula* PCNA homologues. *M. sedula* PCNA homologues were pulled down by His_6_-tagged MsePCNA1 (upper panel), His_6_-tagged MsePCNA2 (middle panel) or His_6_-tagged MsePCNA3 (lower panel) using Co^2+^-immobilised resin. Proteins bound to the resin were eluted with 500 mM imidazole and analysed by SDS-PAGE. The left lane is a molecular weight marker showing the molecular weight of 35,000 and 25,000.

A His_6_-tag mediated pull-down assay was carried out to specify the interactions among the *M. sedula* PCNA homologues (Fig. 2). His_6_-tagged MsePCNA1 and His_6_-tagged MsePCNA2 pulled down MsePCNA2 and MsePCNA1, respectively, indicating an interaction between MsePCNA1 and MsePCNA2. MsePCNA3 was not pulled down by His_6_-tagged MsePCNA1 and His_6_-tagged MsePCNA2 individually, but was pulled down by His_6_-tagged MsePCNA1 with MsePCNA2 and by His_6_-tagged MsePCNA2 with MsePCNA1. Moreover, His_6_-tagged MsePCNA3 pulled down MsePCNA1 and MsePCNA2 together. These results suggest that MsePCNA1 and MsePCNA2 form a heterodimer complex, and then MsePCNA3 binds to the complex.

### Evaluation of heterotrimerisation of *M. sedula* PCNA homologues by size exclusion chromatography

**Figure 3:**
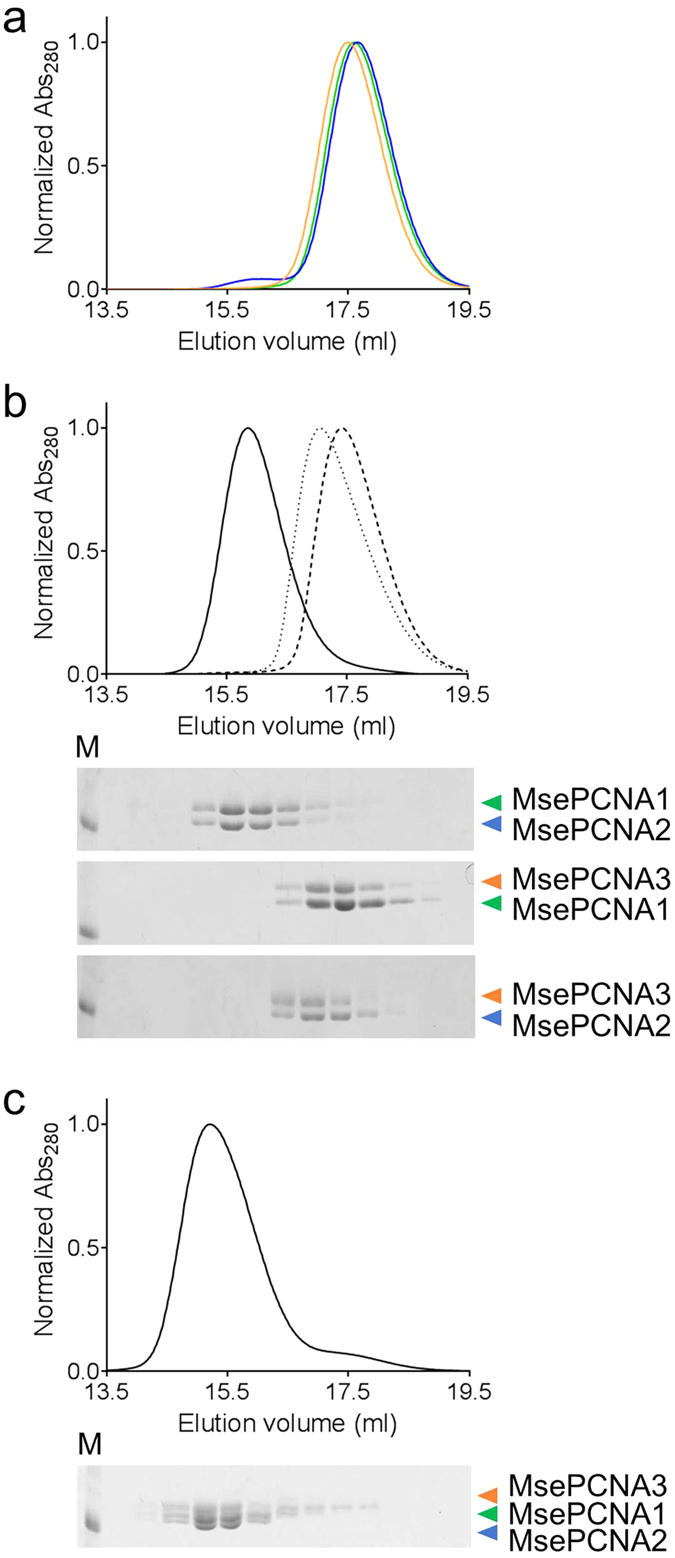
Size exclusion chromatography analysis of the *M. sedula* PCNA homologues. (**a**) Elution profiles of MsePCNA1 (green), MsePCNA2 (blue) and MsePCNA3 (orange) from a Superdex 200 10/300 GL column. (**b**) Elution profiles of equimolar (120 μM) mixtures of MsePCNA1 and MsePCNA2 (solid line), MsePCNA1 and MsePCNA3 (broken line), and MsePCNA2 and MsePCNA3 (dotted line) from the column. Elution fractions were analysed by SDS-PAGE (upper gel, MsePCNA1 and MsePCNA2; middle gel, MsePCNA1 and MsePCNA3; bottom gel, MsePCNA2 and MsePCNA3). (**c**) Elution profile of an equimolar (120 μM) mixture of MsePCNA1, MsePCNA2 and MsePCNA3. The bottom panel shows SDS-PAGE analysis of the elution fractions. The band in the molecular weight maker lane (M) indicates 35,000.

We analysed the complex formation of the PCNA homologues using size exclusion chromatography. MsePCNA1, MsePCNA2 and MsePCNA3 showed a single peak each (Fig. 3a), which was estimated to be around 30 kDa (Supplementary Table 3). An equimolar mixture of MsePCNA1 and MsePCNA2 showed an earlier peak, which was estimated to be 71 kDa, than MsePCNA1 or MsePCNA2 (Fig. 3b). Its elution volume was similar to that of the heterodimer composed of SsoPCNA1 and SsoPCNA2 (Supplementary Fig. S2). This finding indicates that MsePCNA1 and MsePCNA2 form a heterodimer. In contrast, a mixture containing MsePCNA1 and MsePCNA3, or MsePCNA2 and MsePCNA3 showed a peak corresponding to their monomers. This result is consistent with the fact that interactions between MsePCNA1 and MsePCNA3, and between MsePCNA2 and MsePCNA3 were not detected by the pull-down assays. An equimolar mixture of MsePCNA1, MsePCNA2 and MsePCNA3 showed an earlier peak, which contained all the three proteins, than an equimolar mixture of MsePCNA1 and MsePCNA2 (Fig. 3c). Its elution volume was similar to that of the heterotrimer composed of SsoPCNA1, SsoPCNA2 and SsoPCNA3 (Supplementary Fig. S2). Therefore, MsePCNA1, MsePCNA2 and MsePCNA3 form a heterotrimer in a stepwise manner: first MsePCNA1 and MsePCNA2 form a heterodimer, and then MsePCNA3 forms a heterotrimer with the heterodimer, as reported for *S. solfataricus* PCNAs^28^.

### *M. sedula* PCNA homologues interact with each other in a head-to-tail fashion

The above pull-down assay and size exclusion chromatography analysis revealed that MsePCNA1, MsePCNA2 and MsePCNA3 form a heterotrimer in a stepwise fashion. However, these analyses cannot reveal whether the three proteins interact with each other in a head-to-tail fashion. To identify the interacting domains, the interactions among the three proteins were evaluated using the Förster resonance energy transfer (FRET) phenomenon. In the *S. solfataricus* PCNA heterotrimer, all termini of the subunits are located close to the interfaces between the subunits (Fig. 1a). The distance between the C-terminus of SsoPCNA1 and the N-terminus of SsoPCNA2, the C-terminus of SsoPCNA2 and the N-terminus of SsoPCNA3, and the C-terminus of SsoPCNA3 and the N-terminus of SsoPCNA1 is approximately 35 Å. In contrast, the distance between the C-terminus of SsoPCNA1 and the N-terminus of SsoPCNA3, the C-terminus of SsoPCNA2 and the N-terminus of SsoPCNA1, and the C-terminus of SsoPCNA3 and the N-terminus of SsoPCNA2 is more than 70 Å. The Förster distance between a yellow fluorescent protein and a cyan fluorescent protein at which the energy transfer efficiency is 50% is approximately 50 Å^41^. Therefore, FRET from the cyan fluorescent protein, CyPet, fused at the C-terminus of a PCNA subunit, to the yellow fluorescent protein, YPet, fused at the N-terminus of the other PCNA subunit, can reveal whether the C-terminal domain is adjacent to the N-terminal domain.

**Figure 4:**
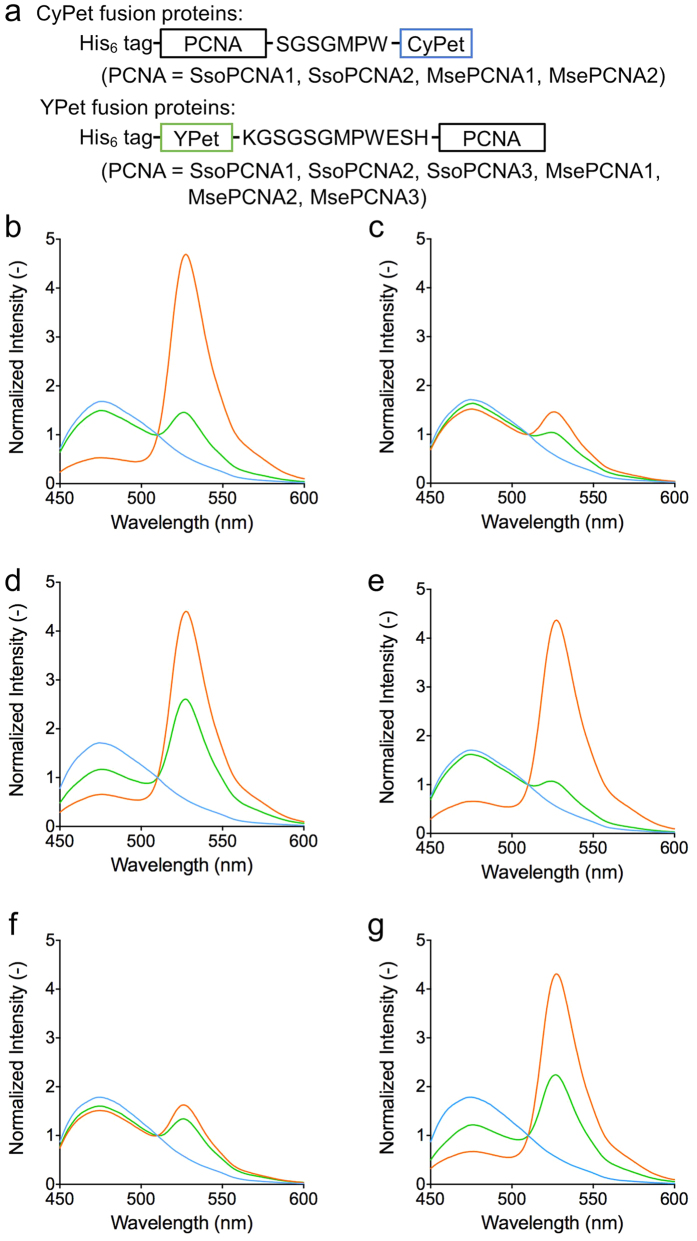
FRET-based analysis to identify interactions between the domains of the *M. sedula* PCNA homologues. (**a**) Constructs of fusion proteins. CyPet was fused to the C-termini of SsoPCNA1, SsoPCNA2, MsePCNA1 and MsePCNA2. YPet was fused to the N-termini of SsoPCNA1, SsoPCNA2, SsoPCNA3, MsePCNA1, MsePCNA2 and MsePCNA3. (**b–g**) Emission spectra of protein mixtures excited at 400 nm. FRET phenomena were observed in equimolar mixtures of (**b**) SsoPCNA1-CyPet and YPet-SsoPCNA2, (**c**) YPet-SsoPCNA1 and SsoPCNA2-CyPet, (**d**) SsoPCNA1, SsoPCNA2-CyPet and YPet-SsoPCNA3, (**e**) MsePCNA1-CyPet and YPet-MsePCNA2, (**f**) YPet-MsePCNA1 and MsePCNA2-CyPet, and (**g**) MsePCNA1, MsePCNA2-CyPet and YPet-MsePCNA3 (orange). Also, emission spectra of the mixtures to which 5 μM (**b**) SsoPCNA2, (**c**) SsoPCNA1, (**d**) SsoPCNA3, (**e**) MsePCNA2 and (**g**) MsePCNA3, and (**f**) 10 μM MsePCNA1 were added, respectively, were measured (green). Cyan lines show the spectra of CyPet fusion proteins in the respective mixtures. The emission spectra were normalised at 510 nm.

First, we confirmed that domain-domain interactions in the *S. solfataricus* PCNA heterotrimer can be identified by the FRET between the fluorescent proteins fused to the PCNAs (Fig. 4a). The mixture of SsoPCNA1-CyPet and YPet-SsoPCNA2 showed a high FRET signal (I_528_/I_477_), which decreased upon addition of SsoPCNA2 (Fig. 4b). In contrast, the mixture of YPet-SsoPCNA1 and SsoPCNA2-CyPet showed a low FRET signal (Fig. 4c). A high FRET signal was also observed with the mixture of SsoPCNA1, SsoPCNA2-CyPet and YPet-SsoPCNA3 (Fig. 4d). Therefore, interaction between the C-terminal domain of a PCNA subunit fused to CyPet and the N-terminal domain of another PCNA subunit fused to YPet induces a high FRET signal.

Next, the interactions between the domains of the *M. sedula* PCNA homologues were evaluated by FRET. The mixture of MsePCNA1-CyPet and YPet-MsePCNA2 showed a high FRET signal, which decreased upon addition of an excess amount of MsePCNA2 (Fig. 4e). In contrast, the mixture of YPet-MsePCNA1 and MsePCNA2-CyPet showed a low FRET signal (Fig. 4f). These results indicate that the C-terminal domain of MsePCNA1 directly interacts with the N-terminal domain of MsePCNA2. In the same manner, an equimolar mixture of MsePCNA1, MsePCNA2-CyPet and YPet-MsePCNA3 showed a high FRET signal, which decreased upon addition of an excess amount of MsePCNA3 (Fig. 4g). Therefore, the N-terminus of MsePCNA3 interacts with the C-terminus of MsePCNA2 in the presence of MsePCNA1. Based on the above findings, it is clear that MsePCNA1, MsePCNA2 and MsePCNA3 form the heterotrimer in a head-to-tail fashion.

### Surface plasmon resonance analysis of the interactions among the *M. sedula* PCNA homologues

The interactions among the *M. sedula* PCNA homologues were quantitatively evaluated by surface plasmon resonance (SPR) analysis. MsePCNA3 did not interact with the immobilised MsePCNA2 on the sensor chip, while MsePCNA1 rapidly associated with the immobilised MsePCNA2 on the sensor chip and dissociated extremely slowly (Supplementary Fig. S3). As a result, the dissociation constant (*K*_d_) of MsePCNA1 and MsePCNA2 was 0.33 ± 0.12 nM (Table 1). This interaction between MsePCNA1 and MsePCNA2 is comparable to that between SsoPCNA1 and SsoPCNA2, probably because the exposed residues on the interface between SsoPCNA1 and SsoPCNA2 are well conserved in MsePCNA1 and MsePCNA2 (Fig. 1b).

The slow dissociation of MsePCNA1 enabled evaluation of the interaction between MsePCNA3 and the MsePCNA1:MsePCNA2 heterodimer on the sensor chip. The *K*_d_ of MsePCNA3 could not be determined kinetically, because of the rapid association and dissociation of MsePCNA3, but it was determined to be 43 ± 3 nM from the plateaued SPR signals at various concentrations of MsePCNA3 (Supplementary Fig. S4). This value is one order of magnitude lower than the *K*_d_ of SsoPCNA3 and the SsoPCNA1:SsoPCNA2 heterodimer (2.0 × 10^2^ nM), which was previously determined to be 2.7 × 10^2^ nM using a GST-fused SsoPCNA1-immobilised sensor chip^28^. However, the residues exposed on the interfaces between SsoPCNA2 and SsoPCNA3, and between SsoPCNA3 and SsoPCNA1 are well conserved in the *M. sedula* PCNA homologues (Fig. 1b). Therefore, the *M. sedula* PCNA homologues may have more appropriate conformations than the SsoPCNAs for interactions such as hydrogen bonds, electrostatic interactions and hydrophobic interactions between the residues on the interfaces.

### Stimulation of DNA ligase 1 activity

**Figure 5:**
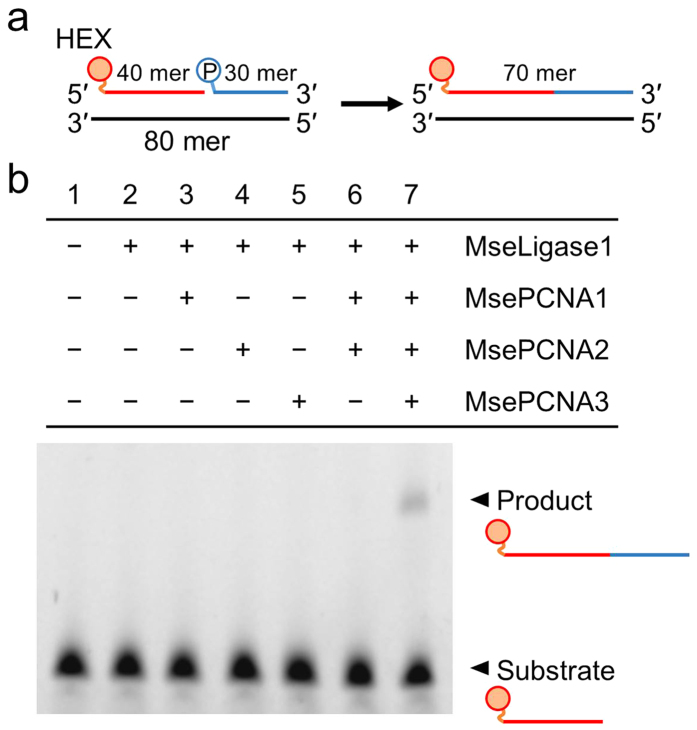
Stimulation of DNA ligase 1 activity by *M. sedula* PCNA homologues. (**a**) Hexachlorofluorescein (HEX)-labelled DNA (30 mer) ligated with 5′ phosphorylated DNA (40 mer) on a template DNA (80 mer). (**b**) Denatured polyacrylamide gel electrophoresis analysis of the ligation reaction in the presence of *M. sedula* PCNA homologue(s).

We examined whether *M. sedula* PCNA homologues stimulate DNA ligase 1 activity, which is involved in DNA replication and DNA repair processes^42^. The *M. sedula* homologue of ATP-dependent DNA ligase 1 (Msed_0150, UniProt: A4YD25), MseLig1, has 69% sequence identity with *S. solfataricus* DNA ligase 1, which is known to be stimulated in the presence of the *S. solfataricus* PCNA heterotrimer^28^. The nick closing activity of MseLig1 (Fig. 5a) was not stimulated by MsePCNA1, MsePCNA2, MsePCNA3 or the mixture of MsePCNA1 and MsePCNA2, but was stimulated by the presence of the three *M. sedula* PCNA homologues together (Fig. 5b). This result indicates that the heterotrimer of MsePCNA1, MsePCNA2 and MsePCNA3 works as a DNA sliding clamp.

### *M. sedula* PCNA homologues interact with *S. solfataricus* PCNAs

The *M. sedula* PCNA homologues were expected to interact with the *S. solfataricus* PCNAs, because the exposed residues on the interface between the *S. solfataricus* PCNAs in the heterotrimer are well conserved in the *M. sedula* PCNA homologues (Supplementary Fig. S1). To examine the interactions among the *M. sedula* PCNA homologues and the *S. solfataricus* PCNAs, a pull-down assay using His_6_-tagged *S. solfataricus* PCNAs was performed. His_6_-tagged SsoPCNA1 pulled down MsePCNA2, and SsoPCNA3 with MsePCNA2 (Supplementary Fig. S5a). His_6_-tagged SsoPCNA2 pulled down MsePCNA1, and SsoPCNA3 with MsePCNA1 (Supplementary Fig. S5b). These observations suggest that MsePCNA1 and MsePCNA2 can substitute SsoPCNA1 and SsoPCNA2, respectively. Unfortunately, interactions of MsePCNA3 with SsoPCNA1 and SsoPCNA2 could not be evaluated, because MsePCNA3 was not separated from His_6_-tagged SsoPCNA1 and His_6_-tagged SsoPCNA2 by SDS-PAGE.

### Identification of interaction domains of the *M. sedula* PCNA homologues with the *S. solfataricus* PCNAs

**Figure 6:**
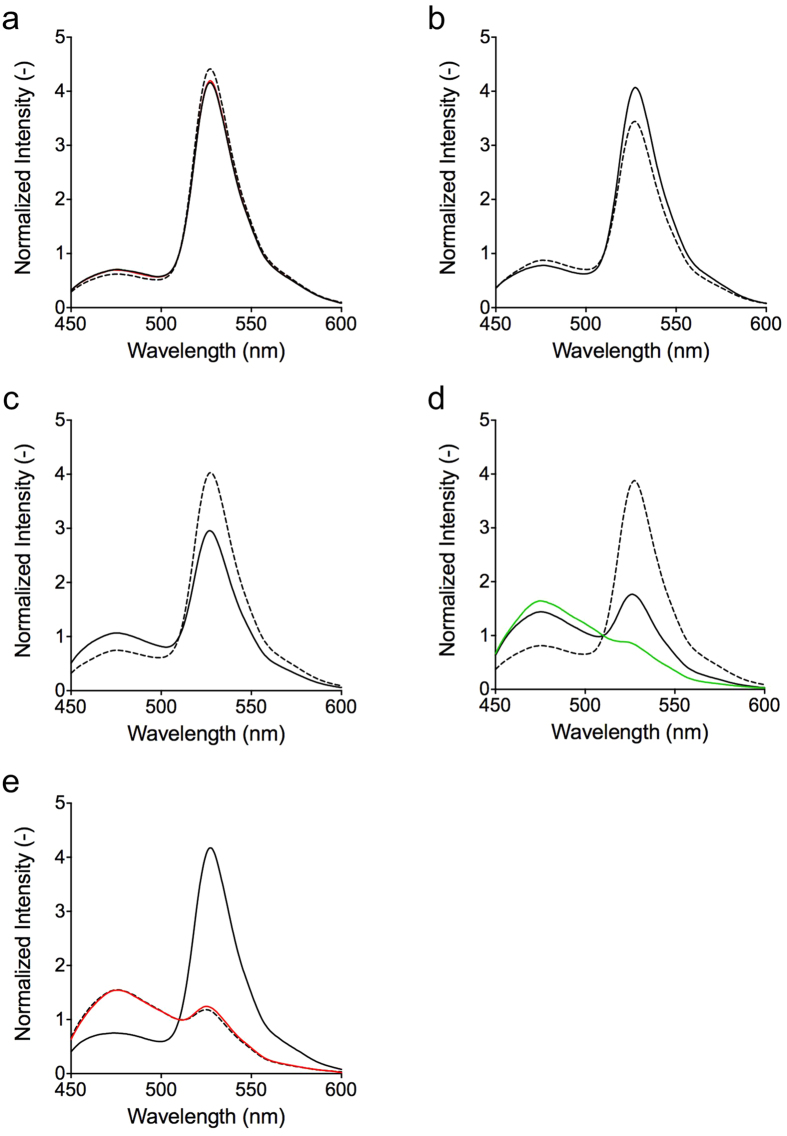
FRET-based analysis of interactions between domains of the *S. solfataricus* PCNAs and the *M. sedula* PCNA homologues. Emission spectra of equimolar mixtures of (**a**) MsePCNA1-CyPet and YPet-SsoPCNA2 (black solid line), and SsoPCNA1-CyPet and YPet-MsePCNA2 (black broken line), (**b**) MsePCNA1, SsoPCNA2-CyPet and YPet-SsoPCNA3 (solid line), and SsoPCNA1, MsePCNA2-CyPet and YPet-MsePCNA3 (broken line), (**c**) MsePCNA1, SsoPCNA2-CyPet and YPet-MsePCNA3 (solid line), and SsoPCNA1, MsePCNA2-CyPet and YPet-SsoPCNA3 (broken line), (**d**) SsoPCNA1, SsoPCNA2-CyPet and YPet-MsePCNA3 (black solid line), and MsePCNA1, MsePCNA2-CyPet and YPet-SsoPCNA3 (black broken line), and (**e**) YPet-MsePCNA1 and SsoPCNA2-CyPet (black solid line), and YPet-SsoPCNA1 and MsePCNA2-CyPet (black broken line). (**a,e**) Emission spectra of a mixture containing 100 μM SsoPCNA3 in addition to MsePCNA1-Cypet and Ype-SsoPCNA2 (**a**), and to Ypet-MsePCNA1 and SsoPCNA2-Cypet (**e**) were measured (red line). A green line shows the emission spectra of an equimolar mixture of SsoPCNA1, SsoPCNA2-CyPet, YPet-MsePCNA3 and MsePCNA3. The emission spectra excited at 400 nm were normalised at 510 nm.

We further evaluated the interactions among the *S. solfataricus* PCNAs and the *M. sedula* PCNA homologues by FRET. High FRET signals were observed in equimolar mixtures of MsePCNA1-CyPet and YPet-SsoPCNA2, and SsoPCNA1-CyPet and YPet-MsePCNA2 (Fig. 6a), indicating interactions between the C-terminal domain of MsePCNA1 and the N-terminal domain of SsoPCNA2, and between the C-terminal domain of SsoPCNA1 and the N-terminal domain of MsePCNA2. High FRET signals were also observed in equimolar mixtures of MsePCNA1, SsoPCNA2-CyPet and YPet-SsoPCNA3, and SsoPCNA1, MsePCNA2-CyPet and YPet-MsePCNA3 (Fig. 6b), indicating that MsePCNA1 and SsoPCNA1 induce interactions between the C-terminal domain of SsoPCNA2 and the N-terminal domain of SsoPCNA3, and between the C-terminal domain of MsePCNA2 and the N-terminal domain of MsePCNA3, respectively. The interactions between the C-terminal domain of MsePCNA2 and the N-terminal domain of SsoPCNA3, and between the C-terminal domain of SsoPCNA2 and the N-terminal domain of MsePCNA3 were confirmed by high FRET signals of mixtures containing SsoPCNA1, MsePCNA2-CyPet and YPet-SsoPCNA3, and MsePCNA1, SsoPCNA2-CyPet and YPet-MsePCNA3, respectively (Fig. 6c). While an equimolar mixture of MsePCNA1, MsePCNA2-CyPet and YPet-SsoPCNA3 demonstrated a high FRET signal (Fig. 6d), that of SsoPCNA1, SsoPCNA2-Cypet and YPet-MsePCNA3 demonstrated a relatively low FRET signal (Fig. 6d) that decreased in the presence of MsePCNA3 (Fig. 6d), indicating a low affinity of MsePCNA3 for the SsoPCNA1:SsoPCNA2 heterodimer. Nonetheless, the FRET analysis suggests that MsePCNA1, MsePCNA2 and MsePCNA3 can form complexes with the *S. solfataricus* PCNAs in a head-to-tail fashion as substitutes for SsoPCNA1, SsoPCNA2 and SsoPCNA3, respectively.

Although the C-terminal domain of MsePCNA1 and the N-terminal domain of SsoPCNA2 were shown to interact with each other, an equimolar mixture of YPet-MsePCNA1 and SsoPCNA2-CyPet unexpectedly showed a high FRET signal (Fig. 6e). In contrast, that of YPet-SsoPCNA1 and MsePCNA2-CyPet showed a low FRET signal. This result suggests that there is an interaction between the N-terminal domain of MsePCNA1 and the C-terminal domain of SsoPCNA2 in addition to the interaction between the C-terminal domain of MsePCNA1 and the N-terminal domain of SsoPCNA2. Considering the fact that the N-terminal domain of SsoPCNA3 interacted with the C-terminal domain of SsoPCNA2 in the presence of MsePCNA1 (Fig. 6b), the N-terminal domain of MsePCNA1 should compete with that of SsoPCNA3 for the interaction with the C-terminal domain of SsoPCNA2. Indeed, addition of SsoPCNA3 decreased the FRET signal between YPet-MsePCNA1 and SsoPCNA2-CyPet (Fig. 6e, red line), but did not affect the FRET signal between MsePCNA1-Cypet and Ypet-SsoPCNA2 (Fig. 6a, red line). Two PCNAs from *S. tokodaii* have been reported to form a ring-shaped tetramer, comprising a homodimer of a heterodimer, where the subunits interact with each other in a head-to-tail manner^33^. Therefore, MsePCNA1 and SsoPCNA2 may form homooligomers of a heterodimer such as a heterotetramer in a head-to-tail fashion and SsoPCNA3 may competitively interact with the N-terminal domain of MsePCNA1 and the C-terminal domain of SsoPCNA2 to form the heterotrimer, MsePCNA1:SsoPCNA2:SsoPCNA3.

### Size exclusion chromatography analysis of a hetero-complex consisting of MsePCNA1 and SsoPCNA2

**Figure 7:**
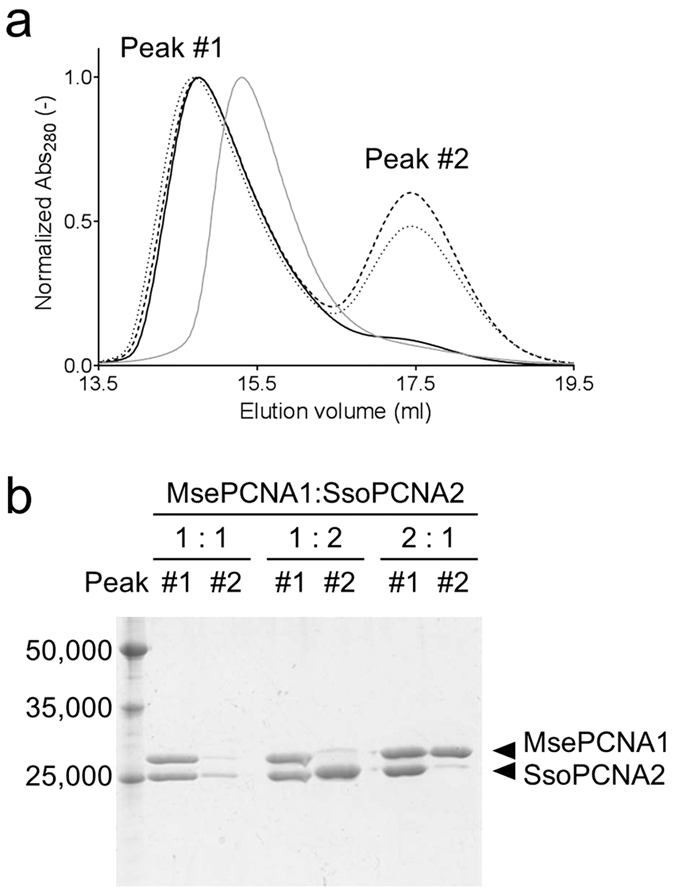
Size exclusion chromatography analysis of mixtures of the MsePCNA1 and SsoPCNA2. (**a**) Elution profiles of mixtures containing MsePCNA1 and SsoPCNA2 in ratios of 1:1 (solid line), 1:2 (broken line) or 1:2 (dotted line), and of the MsePCNA1:MsePCNA2:MsePCNA3 heterotrimer (grey) from the Superdex 200 10/300 GL column. (**b**) SDS-PAGE analysis of the elution peaks. The left lane is a molecular weight marker containing 50,000, 35,000 and 25,000.

To verify that MsePCNA1 and SsoPCNA2 form a homooligomer(s) of a heterodimer, the mixtures containing MsePCNA1 and SsoPCNA2 in molar ratios of 1:1, 2:1 and 1:2 were analysed by size exclusion chromatography (Fig. 7). The equimolar mixture showed a single earlier elution peak, which contained equimolar amounts of MsePCNA1 and SsoPCNA2, than the MsePCNA1:MsePCNA2:MsePCNA3 heterotrimer. The mixtures containing MsePCNA1 and SsoPCNA2 in ratios of 2:1 and 1:2 demonstrated two elution peaks; the former peaks were identical to the elution peak of the equimolar mixture of MsePCNA1 and SsoPCNA2, and the latter peaks contained excess monomers in the respective mixtures. These results suggest that MsePCNA1 and SsoPCNA2 form a heterotetramer composed of two molecules each of MsePCNA1 and SsoPCNA2, but not a heterotrimer composed of one molecule of MsePCNA1 and two molecules of SsoPCNA2, or two molecules of MsePCNA1 and one molecule of SsoPCNA2, as reported for *S. tokodaii* PCNAs^33^.

### SPR analysis to determine the dissociation constants of the *M. sedula* PCNA homologues and the *S. solfataricus* PCNAs

The *K*_d_ values of the *M. sedula* PCNA homologues and the *S. solfataricus* PCNAs were determined by SPR analysis. SsoPCNA1 dissociated from the immobilised MsePCNA2 faster than MsePCNA1, and as a result the *K*_d_ value between SsoPCNA1 and MsePCNA2 was higher than that between MsePCNA1 and MsePCNA2 (Table 1). The immobilised MsePCNA2 that tethered SsoPCNA1 interacted with SsoPCNA3 and MsePCNA3, and the *K*_d_ values were similar to that of the *S. solfataricus* PCNA heterotrimer.

MsePCNA1 dissociated from the immobilised SsoPCNA2 faster than SsoPCNA1, and the *K*_d_ value between MsePCNA1 and SsoPCNA2 was similar to that between SsoPCNA1 and MsePCNA2. Though the FRET analysis suggested an interaction of SsoPCNA3 or MsePCNA3 with the MsePCNA1:SsoPCNA2 complex, the SPR signal did not increase significantly when SsoPCNA3 or MsePCNA3 was flowed on the sensor chip immobilising SsoPCNA2 that tethered MsePCNA1 (Supplementary Fig. S6). This may be because of the weak interaction of SsoPCNA3 or MsePCNA3 with the MsePCNA1:SsoPCNA2 complex. In fact, a higher amount of SsoPCNA3 (up to 3 μM) was not sufficient to determine the dissociation constant (Supplementary Fig. S7).

SsoPCNA3 bound to the immobilised MsePCNA2 with MsePCNA1 (Supplementary Fig. S8a). In contrast, MsePCNA3 did not bind to the immobilized SsoPCNA2 with SsoPCNA1 (Supplementary Fig. S8b), probably because of the low affinity between MsePCNA3 and the SsoPCNA1:SsoPCNA2 heterodimer as suggested by the FRET analysis. These results indicate that the different stability of each heterotrimer is not mainly due to the amino acids at the interaction sites, but due to other factors such as conformation changes after the formation of heterodimers or heterotrimers, because *S. solfataricus* PCNAs and *M. sedula* PCNA homologues share similar amino acid residues that are exposed at the interfaces.

## Conclusions

We demonstrated that the *M. sedula* PCNA homologues formed a heterotrimer in a stepwise fashion as reported for *S. solfataricus* PCNAs. Though the exposed residues on the interfaces between the subunits in the *S. solfataricus* PCNA heterotrimer are well conserved in the PCNA homologues, the *M. sedula* PCNA homologues formed a more stable heterotrimer than the *S. solfataricus* PCNAs. Lately, protein assembly has been attracting more attention in the field of biotechnology for generating artificial multi-enzyme complexes, functional hydrogels and protein fibres. Stable and stepwise heterotrimerisation of *M. sedula* PCNAs would be beneficial to homogeneously construct artificial protein complexes without complicated procedures. The nick closing activity of *M. sedula* DNA ligase 1 was enhanced by the simultaneous presence of the three PCNA homologues, suggesting the heterotrimer of the PCNA homologues works as a DNA sliding clamp. We also showed that *M. sedula* PCNA homologues and *S. solfataricus* PCNAs could interact with each other, as expected from the high homology between the *M. sedula* PCNA homologues and the *S. solfataricus* PCNAs. However, the affinity between MsePCNA3 and the SsoPCNA1:SsoPCNA2 dimer was much lower than that between MsePCNA3 and the MsePCNA1:MsePCNA2 dimer or between SsoPCNA3 and the SsoPCNA1:SsoPCNA2 dimer. Interestingly, MsePCNA1 and SsoPCNA2 formed a heterotetramer. These observations indicate that assembly of PCNAs is not dominated only by their surface residues. Future structural analysis will reveal the details of the heterogeneous PCNA interactions.

## Methods

### Pull-down assay

A suspension prepared from 30 μl of a 50% slurry of TALON Metal affinity resin (Mountain View, CA, USA), and 60 μl of a mixture containing 30 μM His_6_-tagged protein and 30 μM His_6_-tag-removed protein(s) in a 20 mM potassium phosphate buffer, pH 7.4, containing 150 mM KCl and 10 mM imidazole was incubated on ice for 30 min. Next, the resin was washed twice with 1 ml of the buffer. The proteins tethered to the resin were eluted with 30 μl of the 20 mM potassium phosphate buffer, pH 7.4, containing 150 mM KCl and 500 mM imidazole. The eluted proteins were separated by SDS-PAGE and stained by Coomassie Brilliant Blue R-250.

### Size exclusion chromatography analysis

A mixture containing 120 μM protein(s) in 150 μl of 50 mM potassium phosphate buffer, pH7.4, containing 150 mM KCl was incubated on ice for more than 1 h. Then, the mixture was subjected to size exclusion chromatography on Superdex 200 10/300 GL column (GE Healthcare, Little Chalfont, UK) at a flow rate of 1.0 ml/min with 50 mM potassium phosphate buffer, pH 7.4, containing 150 mM KCl.

### Fluorescence resonance energy transfer-based analysis

Protein concentrations of SsoPCNA1, SsoPCNA2, SsoPCNA3, MsePCNA1, MsePCNA2 and MsePCNA3 were determined spectrophotometrically, using the extinction coefficients of 14.4, 13.4, 13.4, 17.4, 11.9, 8.94 mM^−1^ cm^−1^, respectively, at 280 nm, which were calculated from their amino acid compositions. Those of YPet-fusion proteins and CyPet-fusion proteins were determined spectrophotometrically, using the extinction coefficients of YPet at 514 nm (ε_514_ = 104 mM^−1^ cm^−1^) and CyPet at 433 nm (ε_433_ = 35 mM^−1^ cm^−1^), respectively^43^. PCNA proteins were mixed at a final concentration of 1 μM in 90 μL of 50 mM potassium phosphate buffer, pH7.4, containing 150 mM KCl and incubated on ice for 15 min. Then, the mixtures were excited at 400 nm and emission spectra were measured. The emission spectra were normalised at 510 nm. For the competitive analysis, MsePCNAs or SsoPCNAs were added to the protein mixtures at a final concentration of 5, 10 or 100 μM.

### SPR analysis

According to the Biacore (GE Healthcare) protocols, about 350 RU of MsePCNA2 or SsoPCNA2 were immobilised on Sensor Chip CM5. Then, the sensorgram was monitored by injecting various concentrations of MsePCNA1 or SsoPCNA1. At the end of each cycle bound proteins were removed by washing with 10 mM glycine-HCl buffer, pH 1.5, at a rate of 10 ml/min for quantitative analysis and at a rate of 20 μl/min for kinetics analysis. The kinetics values were determined from association and dissociation curves of the sensorgrams, using BIAevaluation software. To determine the dissociation constants of MsePCNA3 and SsoPCNA3 from protein complexes formed on the sensor chip, various concentrations of MsePCNA3 or SsoPCNA3 were injected following application of 1 μM MsePCNA1 or SsoPCNA1. To eliminate the effect of the dissociation of MsePCNA1 or SsoPCNA1, the sensorgram obtained without injecting MsePCNA3 or SsoPCNA3 was subtracted from each sensorgram. As a result, the effective increase in the SPR signal by the binding of MsePCNA3 or SsoPCNA3 to a protein complex coupled to the sensor chip was determined. The dissociation constants were determined by plotting the increase in the SPR signal against the injected protein concentration followed by fitting response to Langmuir adsorption isotherm using Prism6 (GrapPad Software, La Jolla, CA, USA). All the measurements were performed in 10 mM HEPES-HCl buffer, pH 7.4, with 150 mM NaCl.

### Nick closing assay

A mixture containing 10 mM HEPES, pH 8.0, 10 mM magnesium chloride, 1 mM ATP, 300 nM *M. sedula* DNA ligase 1, 1 μM *M. sedula* PCNA homologues, 1 μM 5′ phosphorylated DNA (5′-CAGAGGATTGTTGACCGGCCCGTTTGTCAG-3′), 1 μM 5′ hexachlorofluorescein (HEX)-labelled DNA (5′-CGCACCGTGACGCCAAGCTTGCATTCCTACAGGTCGACTC-3′) and 1 μM template DNA (5′-CGTTGCTGACAAACGGGCCGGTCAACAATCCTCTGGAGTCGACCTGTAGGAATGCAAGCTTGGCGTCACGGTGCGCCAAC-3′) was incubated at 50 °C for 90 min. Then, 10 μl of loading solution containing 98% formamide, 10 mM EDTA, bromophenol blue and xylen cyanol were added to 10 μl of the reaction mixture. After boiling for 5 min, 6 μl of the mixture were separated on 10% 8 M-urea polyacrylamide gel.

## Additional Information

**How to cite this article**: Iwata, F. *et al.* Three proliferating cell nuclear antigen homologues from *Metallosphaera sedula* form a head-to-tail heterotrimer. *Sci. Rep.* **6**, 26588; doi: 10.1038/srep26588 (2016).

## Supplementary Material

Supplementary Information

## Figures and Tables

**Table 1:** Binding parameters of interactions among the *M. sedula* PCNA homologues and the *S. solfataricus* PCNAs.

Immobilised protein	Analyte 1	*k*_on_ (M^−1^ s^−1^)^a^	*k*_off_ (s^−1^)^a^	*K*_d_ (M)^b^	*K*_d_ (M) of Analyte 2^c^	
					MsePCNA3	SsoPCNA3
MsePCNA2	MsePCNA1	(2.8 ± 0.6) × 10^5^	(8.9 ± 3.5) × 10^−5^	(3.3 ± 1.2) × 10^−10^	(4.3 ± 0.3) × 10^−8^	(1.9 ± 0.1) × 10^−7^
	SsoPCNA1	(2.7 ± 1.3) × 10^5^	(4.4 ± 1.5) × 10^−4^	(1.8 ± 0.5) × 10^−9^	(3.8 ± 0.1) × 10^−7^	(1.7 ± 0.2) × 10^−7^
SsoPCNA2	MsePCNA1	(1.3 ± 0.8) × 10^5^	(1.2 ± 0.1) × 10^−3^	(1.6 ± 1.3) × 10^−9^	N.D.	N.D.
	SsoPCNA1	(2.5 ± 1.4) × 10^5^	(1.5 ± 0.5) × 10^−4^	(6.5 ± 2.5) × 10^−10^	N.D.	(2.0 ± 0.3) × 10^−7^

^a^The mean and standard error were calculated from the data at 2.5, 5, 10, 20 and 40 nM MsePCNA1 or SsoPCNA1.

^b^The mean and standard error were calculated from values that were independently determined from the *k*_on_ and *k*_off_ at the respective concentrations (Supplementary Table 4).

^c^Error of *K*_d_ values is standard error of the mean determined from the curve fitting of the saturated differential resonance unit against MsePCNA3 or SsoPCNA3 concentration. N.D., not determined.
