# Supplementary material for: Three proliferating cell nuclear antigen homologues from Metallosphaera sedula form a head-to-tail heterotrimer
Source: Sci Rep. 2016 May 27;6:26588. doi: 10.1038/srep26588 (PMC4894655; doi:10.1038/srep26588)
Supplement: Supplementary Information [file srep26588-s1.pdf]

Supplementary Information

Three proliferating cell nuclear antigen homologues from *Metallosphaera sedula* form a head-to-tail heterotrimer

Fumiya Iwata[1], Hidehiko Hirakawa[1,*], Teruyuki Nagamune[1,2]

[1]Department of Chemistry and Biotechnology, School of Engineering, The University of Tokyo, 7-3-1 Hongo, Bunkyo-ku, Tokyo 113-8656, Japan

[2]Department of Bioengineering, School of Engineering, The University of Tokyo, 7-3-1 Hongo, Bunkyo-ku, Tokyo 113-8656, Japan

*Corresponding author

Tel: +81-3-5841-7356; Fax: +81-3-5841-8657; E-mail: hirakawa@bio.t.u-tokyo.ac.jp

Supplementary Methods

Materials

Escherichia coli T7 Express I^q, T7 Express *lysY*/I^q and pMAL-c5E were purchased from New England Biolabs (Ipswich, MA, USA). Biotinylated thrombin and pET-15b(+) were purchased from Merck (Darmstadt, Germany). Synthetic genes encoding the *Metallosphaera sedula* PCNA subunit homologues were purchased from Biomatik (Cambridge, Ontario, Canada). Synthetic genes encoding the *M. sedula* DNA ligase 1 was purchased from Thirmo Fisher scientific (Waltham, MA, USA). HisTrap FF crude column (1.6 × 2.5 cm), HisTrap HP column (1.6 × 2.5 cm), HiTrap Q FF column (1.6 × 2.5 cm), HiTrap Q HP column (1.6 × 2.5 cm), HiLoad 16/600 Superdex 75 pg column (1.6 × 30 cm), Superdex 200 10/300 GL column (1.0 × 30 cm) and Biacore CM5 sensor chip were purchased from GE Healthcare (Little Chalfont, Buckinghamshire, UK). In-Fusion Cloning Kit and TALON Metal affinity resin were purchased from Clontech (Mountain View, CA, USA). T-vector pMD20 TA was purchased from Takara Bio (Shiga, Japan). Ampicillin (Amp), glucose, isopropyl-β-D-thiogalactoside (IPTG) and Coomassie Brilliant Blue R-250 were purchased from Wako Pure Chemical Industries (Osaka, Japan).

Vector construction

The synthetic genes encoding, Msed_0051 (UniProt: A4YCS6), Msed_1792 (UniProt: A4YHP5) and Msed_2250 (UniProt: A4YIY6), were cloned into pET15T[1] between the *Nde*I and *Bam*HI sites, to generate the plasmids pET15T-MsePCNA1, pET15T-MsePCNA2 and pET15T-MsePCNA3, respectively. The genes encoding SsoPCNA1, SsoPCNA2 and SsoPCNA3, which are cloned into pHSG398[2], were inserted between the *Nde*I and *Bam*HI sites of pET15T, to generate pET15T-SsoPCNA1, pET15T-SsoPCNA2 and pET15T-SsoPCNA3, respectively.

pET15T-SsoPCNA3 was digested with *Nco*I and *Bam*HI, and then the generated DNA fragment was cloned into pMAL-c5E, to generate pMAL-SsoPCNA3. An expression plasmid of the S219V mutant of the Tobacco etch virus (TEV) protease, pMal-TEV, was constructed, as previously described[3], except for using pMAL-c4E as a host vector. The synthetic genes encoding, Msed_0150 (UniProt: A4YD25) was cloned into pET28b between the *Nde*I and *Bam*HI sites, to generate the plasmids pET28b-MseLig1.

A modified pET-15b vector, which lacked the *Nco*I site and harboured a stop codon downstream of the *Bam*HI site, was linearised by PCR using two primers, 5′-AAGCTCGAGGA TCCGTAAGCTAAC-3′ (forward) and 5′-CACCATCATATGGCTGCCGCGC-3′ (reverse). The YPet and CyPet genes were amplified by PCR, and then fused to the linearised plasmid at the same time using In-Fusion Cloning Kit, to obtain pET15b-YC. The Msed_0051 gene was amplified by PCR and cloned into pMD20 according to the manufacturer's protocol. The resulting plasmid, pMD-MsePCNA1, was digested with *Nco*I and *Bam*HI. The generated DNA fragment was ligated into the *Nco*I and *Bam*HI sites of pET15b-YC, to obtain pET15b-YM1, which expresses the YPet-MsePCNA1 fusion protein. The Msed_1792 gene, the Msed_2250 gene and the genes encoding SsoPCNA1, SsoPCNA2 and SsoPCNA3 were amplified by PCR and cloned into pET15b-YC, to obtain the YPet-MsePCNA2, YPet-MsePCNA3, YPet-SsoPCNA1, YPet-SsoPCNA2 and YPet-SsoPCNA3 expression plasmids as described above. The primers used to amplify the genes are summarised in Table S5. The MsePCNA1-CyPet expression plasmid was prepared by inserting a DNA fragment that was generated by digestion of pMD+MsePCNA1 with *Nde*I and *Nco*I, between the *Nde*I and *Nco*I sites of pET15b-YC. The MsePCNA2-CyPet, MsePCNA3-CyPet, SsoPCNA1-CyPet, SsoPCNA2-CyPet and SsoPCNA3-CyPet expression plasmids were also prepared by

inserting DNA fragments that were generated by digestion of the pMD20 vectors harbouring the Msed_1792 gene, the Msed_2250 gene and the genes encoding SsoPCNA1, SsoPCNA2 and SsoPCNA3, between the NdeI and NcoI sites of pET15b-YC, respectively.

Expression and purification

$(His)_6$-MsePCNA1 was expressed in *E. coli* T7 Express *I*q transformed with pET15T-MsePCNA1. A single colony of cells was inoculated into 5 ml of LB medium containing 100 mg/l Amp and 1% glucose, and cultured at 37°C until the OD at 660 nm reached 1.0. Then, the culture was added to 1 l of TB medium containing 100 mg/l Amp and the cells were grown at 37°C until the OD at 600 nm reached 0.7. After 1 mmol of IPTG was added, the culture was incubated at 27°C overnight. The cells were harvested by centrifugation at 6,000 × *g* for 20 min and resuspended in 20 mM potassium phosphate buffer, pH 7.4, containing 150 mM KCl and 10 mM imidazole. After the cells were disrupted by ultrasonication, the cell debris was removed by centrifugation at 22,000 × *g* for 30 min. The resulting cell lysate was incubated at 50°C for 30 min. After removing precipitants by centrifugation at 22,000 × *g* for 30 min, the supernatant was loaded onto a HisTrap FF crude column. After washing the column with the above buffer, $(His)_6$-MsePCNA1 was eluted with a linear gradient of imidazole (10–500 mM). Fractions containing the protein were combined and dialysed against 20 mM potassium phosphate buffer, pH 7.4, containing 150 mM KCl in the presence of TEV protease, which was expressed in *E. coli* BL21 Star (DE3) pLysSRARE[4] and purified as previously described[3]. After the dialysed protein solution was incubated at 50°C for 30 min, the precipitants were removed by centrifugation at 6,000 × *g* for 30 min. The supernatant was loaded onto a HisTrap HP column. The unbound protein was collected as MsePCNA1, while the eluted fractions with 20 mM potassium phosphate buffer, pH 7.4, containing 150 mM KCl and 500

mM imidazole were collected as (His)$_6$-MsePCNA1. After concentration with an Amicon Ultra-15 Centrifugal Unit (10,000 NMWL), MsePCNA1 and (His)$_6$-MsePCNA1 were further purified by size exclusion chromatography using a HiLoad 16/600 Superdex 75 pg column with 50 mM potassium phosphate buffer, pH7.4, containing 150 mM KCl. The purified MsePCNA1 and (His)$_6$-MsePCNA1 were concentrated with the above centrifugal unit. MsePCNA2 and His$_6$-MsePCNA2 were obtained as described above, except that His$_6$-MsePCNA2 eluted from a HisTrap FF crude column was subjected to buffer exchange using a HisTrap Desalting column pre-equilibrated with 20 mM potassium phosphate buffer, pH 7.4, containing 150 mM KCl and 1 mM DTT, and incubated with TEV protease at 30°C for 24 h.

(His)$_6$-MsePCNA3 was expressed and partially purified using a HisTrap FF crude column as described for MsePCNA1. The eluted protein was subjected to size exclusion chromatography on a HiLoad 16/600 Superdex 75 pg column with 50 mM potassium phosphate buffer, pH7.4, containing 150 mM KCl. The purified (His)$_6$-MsePCNA3 was concentrated with the above centrifugal unit. MsePCNA3 was obtained by passing (His)$_6$-MsePCNA3 treated with biotinylated thrombin through a HisTrap HP column.

(His)$_6$-SsoPCNA1 and (His)$_6$-SsoPCNA2 were expressed and purified as described for (His)$_6$-MsePCNA1. SsoPCNA1 and SsoPCNA2 were obtained as described for MsePCNA1, except that the cell lysate and the dialysed protein solution were incubated at 55°C. A fusion protein of SsoPCNA3 with maltose binding protein (MBP) was expressed from pMAL-SsoPCNA3 using T7 Express *lysY/I*^{q} by inoculating cells in TB medium and cultivating them at 27°C after IPTG induction. SsoPCNA3 was obtained as described for MsePCNA2, except that the protein mixture incubated with TEV protease was passed through a HisTrap HP column, incubated at 55°C for 30

min and passed through an MBPTrap HP column, and then the eluted protein was subjected to size exclusion chromatography. $(His)_6$-SsoPCNA2 was expressed and purified as described for $(His)_6$-MsePCNA3, except that the cell lysate was incubated at 55°C.

The YPet-MsePCNA1 fusion protein was expressed from pET15b-YM1 using T7 Express *lysY/I*^{q} by inoculating cells in TB medium and cultivating them at 16°C after IPTG induction. After being partially purified using a HisTrap FF crude column as described for MsePCNA1, the protein solution was diluted with an equivalence volume of 20 mM potassium phosphate buffer, pH 7.4, and then loaded onto a HiTrap Q FF column. After washing the column with 20 mM potassium phosphate buffer, pH 7.4, containing 75 mM KCl, the protein was eluted with a linear gradient of KCl (75–500 mM). The eluted protein was concentrated with an Amicon Ultra-15 Centrifugal Unit (50,000 NMWL). The concentrated protein was subjected to size exclusion chromatography on a HiLoad 16/600 Superdex 75 pg column with 50 mM potassium phosphate buffer, pH7.4, containing 150 mM KCl. MsePCNA1-CyPet, MsePCNA2-CyPet, YPet-SsoPCNA1 and SsoPCNA1-CyPet fusion proteins were expressed and purified as described above. YPet-MsePCNA2, SsoPCNA2-CyPet, YPet-SsoPCNA2 and YPet-SsoPCNA3 fusion proteins were expressed and purified as described for YPet-MsePCNA1, except that the protein solution was not diluted after being partially purified using a HisTrap FF crude column. The YPet-MsePCNA3 fusion protein was expressed and purified as described for YPet-MsePCNA1, except that the cells were cultivated at 27°C after protein induction and the protein solution was not diluted after being partially purified using a HisTrap FF crude column.

MseLig1 was expressed as described for (His_6)-MsePCNA1 except that the cells were cultured at 20 °C overnight after OD at 600 nm reached 0.7 without addition of IPTG. The cells

were harvested by centrifugation at 6,000 × *g* for 20 min, resuspended with 10 mL of the 20 mM HEPES buffer, pH 8.0, containing 150 mM KCl 10 mM imidazole and 10 mg of lysozyme and then lysed by rotating at R.T. for 20 min. The lysed cells were incubated at 70 °C for 30 min and the cell debris was removed by centrifugation at 22,000 × *g* for 30 min. The supernatant was loaded onto a HisTrap FF crude column. After washing the column with the above buffer, MseLig1 was eluted with a linear gradient of imidazole (10–500 mM). The eluted protein was concentrated with an Amicon Ultra-15 Centrifugal Unit (50,000 NMWL) and subjected to size exclusion chromatography on a Hiload Superdex 200 16/600 pg column with the 10 mM HEPES buffer, pH 7.4 containing 150 mM NaCl.

Tables

Supplementary Table 1. Crenarchaeal PCNA homologue genes.

Organism	PCNA homologues (Gene ID)
Desulfurococcales	
Aeropyrum pernix K1	APE_0162 (1445694), APE_0441.1 (1444627), APE_2182 (1445247)
Aeropyrum camini SY1	ACAM_0128 (17111031), ACAM_0319 (17111098), ACAM_1371 (17110614)
Staphylothermus marinus F1	Smar_0638 (4906646), Smar_1002 (4908072), Smar_1365 (4907431)
Staphylothermus hellenicus DSM 12710	Shell_0153 (9233442), Shell_1079 (9234368), Shell_1459 (9234750)
Ignicoccus hospitalis KIN4/I	Igni_0610 (5562642), Igni_0713 (5562948), Igni_0854 (5562750)
Desulfurococcus kamchatkensis 1221n	DKAM_0325 (7171539), DKAM_1184 (7171265), DKAM_1419 (7171677)
Desulfurococcus mucosus DSM 2162	Desmu_0044 (10152724), Desmu_0731 (10153426), Desmu_1188 (10153903)
Desulfurococcus fermentans DSM 16532	Desfe_0043 (13062371), Desfe_0548 (13062238), Desfe_1295 (13061692)
Thermosphaera aggregans DSM 11486	Tagg_0042 (9165049), Tagg_0664 (9165678), Tagg_0834 (9165850)
Ignisphaera aggregans DSM 17230	Igag_0031 (9715347), Igag_0145 (9715461), Igag_0150 (9715466)
Thermogladius cellulolyticus 1633	TCELL_0305 (13012589), TCELL_0458 (13012752), TCELL_0836 (13013153)
Hyperthermus butylicus DSM 5456	Hbut_0018 (4782105), Hbut_1286 (4781450), Hbut_1645 (4782607)
Pyrolobus fumarii 1A	Pyrfu_0182 (11139819), Pyrfu_1696 (11138885), Pyrfu_1903 (11138242)
Sulfolobales	
Sulfolobus solfataricus P2	SSO0397 (1455536), SSO0405 (1455544), SSO1047 (1454088)
Sulfolobus tokodaii strain7	ST0387 (1458312), ST0397 (1458322), ST0944 (1458909)
Sulfolobus acidocaldarius DSM 639	Saci_0817 (3472653), Saci_1280 (3473081), Saci_0826 (3472661)
Sulfolobus islandicus L.S.2.15	LS215_1271 (7797782), LS215_1826 (7799465), LS215_1834 (7799473)
Metallosphaera sedula DSM 5348	Msed_0051 (5104629), Msed_2250 (5104311), Msed_1792 (5105356)
Metallosphaera cuprina Ar-4	Mcup_0050 (10492247), Mcup_0059 (10494231), Mcup_0441 (10492635)
Acidianus hospitalis W1	Ahos_0720 (10600174), Ahos_0729 (10600183), Ahos_1247 (10600726)
Acidilobales	
Acidilobus saccharovorans 345-15	ASAC_0200 (9498418), ASAC_0222 (9498442), ASAC_0781 (9499016)
Caldisphaera lagunensis DSM 15908	Calag_0208 (14211468), Calag_0394 (14211654), Calag_1393 (14212653)
Fervidicoccales	
Fervidicoccus fontis Kam940	FFONT_0432 (12449505), FFONT_1242 (12450344), FFONT_1095 (12450188)
Thermoproteales	
Pyrobaculum aerophilum IM2	PAE0720 (1465204), PAE3038 (1463798)
Pyrobaculum islandicum DSM 4184	Pisl_0438 (4616310), Pisl_0667 (4617557)
Pyrobaculum calidifontis JCM 11548	Pcal_0990 (4909918), Pcal_2131 (4908249)
Pyrobaculum arsenaticum DSM 13514	Pars_1788 (5055602), Pars_2362 (5055647)
Pyrobaculum sp. 1860	P186_1479 (11594738), P186_2218 (11593822)
Pyrobaculum oguniense TE7	Pogu_0343 (11854124), Pogu_2503 (11856407)
Pyrobaculum neutrophilum V24Sta	Tneu_1682 (6165226), Tneu_1995 (6165868)
Caldivirga maquilingensis IC-167	Cmaq_0042 (5709359), Cmaq_1377 (5709160)
Thermoproteus uzoniensis 768-20	TUZN_0224 (10359771), TUZN_0602 (10360145)
Thermoproteus tenax Kra 1	TTX_0580 (11263580), TTX_0869 (11261763)
Vulcanisaeta distributa DSM 14429	Vdis_1828 (9752773), Vdis_2057 (9753008)
Vulcanisaeta moutnovskia 768-28	VMUT_0269 (10287921), VMUT_0465 (10288117)
Thermofilum pendens Hrk 5	Tpen_0624 (4601411)
Thermofilum sp. 1910b	N186_04475 (16573539)

Supplementary Table 2. Sequence identity (%) between PCNA proteins.

	Msed_1792 (MsePCNA2)	Msed_2250 (MsePCNA3)	SsoPCNA1	SsoPCNA2	SsoPCNA3
Msed_0051 (MsePCNA1)	19	22	41	18	19
Msed_1792 (MsePCNA2)		22	18	53	22
Msed_2250 (MsePCNA3)			20	23	46

Supplementary Table 3. Molecular mass estimation by size exclusion chromatography.

Protein	Estimated molecular mass (kDa)	Calculated molecular mass (kDa)
SsoPCNA3	33	29
Equimolar mixture of SsoPCNA1 and SsoPCNA2	73	-
Equimolar mixture of SsoPCNA1, SsoPCNA2 and SsoPCNA3	93	-
MsePCNA1	30	27
MsePCNA2	29	27
MsePCNA3	32	28
Equimolar mixture of MsePCNA1 and MsePCNA2	71	-
Equimolar mixture of MsePCNA1, MsePCNA2 and MsePCNA3	98	-
Equimolar mixture of MsePCNA1 and SsoPCNA2	125	-

Supplementary Table 4. Kinetic parameters determined by curve fitting to the observed SPR signals.

Immobilised protein	Ligand	Concentration (nM)	k_{on} (M^{-1} s^{-1}) [a]	k_{off} (s^{-1}) [a]	K_d (M)
MsePCNA2	MsePCNA1	40	$(1.69\pm0.06)\times10^{5}$	$(4.29\pm0.18)\times10^{-5}$	2.530×10^{-10}
		20	$(2.69\pm0.07)\times10^{5}$	$(5.79\pm0.16)\times10^{-5}$	2.150×10^{-10}
		10	$(3.22\pm0.04)\times10^{5}$	$(7.99\pm0.13)\times10^{-5}$	2.490×10^{-10}
		5	$(3.48\pm0.02)\times10^{5}$	$(1.10\pm0.01)\times10^{-4}$	3.160×10^{-10}
		2.5	$(3.06\pm0.01)\times10^{5}$	$(1.21\pm0.01)\times10^{-4}$	3.960×10^{-10}
		1.25	$(2.41\pm0.01)\times10^{5}$	$(1.28\pm0.01)\times10^{-4}$	5.310×10^{-10}
	SsoPCNA1	75	$(4.43\pm0.74)\times10^{4}$	$(9.46\pm0.77)\times10^{-5}$	2.140×10^{-9}
		40	$(2.70\pm0.43)\times10^{5}$	$(4.39\pm0.03)\times10^{-4}$	1.630×10^{-9}
		20	$(1.77\pm0.03)\times10^{5}$	$(4.72\pm0.03)\times10^{-4}$	2.670×10^{-9}
		10	$(3.20\pm0.02)\times10^{5}$	$(5.01\pm0.02)\times10^{-4}$	1.570×10^{-9}
		5	$(2.94\pm0.02)\times10^{5}$	$(5.05\pm0.02)\times10^{-4}$	1.710×10^{-9}
		2.5	$(3.63\pm0.02)\times10^{5}$	$(5.12\pm0.02)\times10^{-4}$	1.410×10^{-9}
		0.61	$(4.38\pm0.02)\times10^{5}$	$(5.28\pm0.02)\times10^{-4}$	1.210×10^{-9}
SsoPCNA2	MsePCNA1	50	$(4.54\pm0.95)\times10^{4}$	$(1.25\pm0.02)\times10^{-3}$	2.750×10^{-8}
		40	$(3.16\pm0.66)\times10^{4}$	$(1.14\pm0.01)\times10^{-3}$	3.600×10^{-8}
		20	$(9.15\pm1.23)\times10^{4}$	$(1.180\pm0.01)\times10^{-3}$	1.290×10^{-8}
		10	$(1.80\pm0.07)\times10^{5}$	$(1.26\pm0.01)\times10^{-3}$	7.020×10^{-9}
		5	$(2.08\pm0.02)\times10^{5}$	$(1.32\pm0.01)\times10^{-3}$	6.370×10^{-9}
		2.5	$(1.97\pm0.02)\times10^{5}$	$(1.34\pm0.01)\times10^{-3}$	6.810×10^{-9}
	SsoPCNA1	75	$(5.03\pm0.17)\times10^{4}$	$(3.07\pm0.16)\times10^{-5}$	6.100×10^{-10}
		40	$(1.98\pm0.04)\times10^{5}$	$(1.34\pm0.02)\times10^{-4}$	6.780×10^{-10}
		20	$(1.40\pm0.02)\times10^{5}$	$(1.63\pm0.02)\times10^{-4}$	1.160×10^{-10}
		10	$(2.95\pm0.01)\times10^{5}$	$(1.82\pm0.02)\times10^{-4}$	6.170×10^{-10}
		5	$(2.87\pm0.01)\times10^{5}$	$(1.73\pm0.01)\times10^{-4}$	6.020×10^{-10}
		2.5	$(3.62\pm0.02)\times10^{5}$	$(1.71\pm0.01)\times10^{-4}$	4.730×10^{-10}
		0.61	$(4.49\pm0.03)\times10^{5}$	$(1.72\pm0.01)\times10^{-4}$	3.830×10^{-10}

[a] Error in k_{on} and k_{off} values is standard error of the mean determined from curve fitting of SPR signal.

Supplementary Table 5. Primers used to amplify genes.

Protein	Forward /Reverse	Sequence (5'- -3')
YPet	Forward	AGCCATATGATGGTGAGCAAGGGCG
	Reverse	CATCCATGGCATGCCACTACCCTTCTTGTACAGCTC
CyPet	Forward	GGCATGCCATGGATGGTGAGCAAGGGCG
	Reverse	CGGATCCTCGAGCTTGTACAGCTCGTCCATG
MsePCNA1 (Msed_0051)	Forward	GGCATGCCATGGGAATCCCATATGATGTTCCGTGCAATCTATGG
	Reverse	CATGCCATGGCATGCCGGATCCCGACAGACGCGGAGCAAC
MsePCNA2 (Msed_1792)	Forward	GGCATGCCATGGGAATCCCATATGATGAAATTCAAAGTTATCGACGCG
	Reverse	CATGCCATGGCATGCCGGATCCCGATTCCGCACGCGGTG
MsePCNA3 (Msed_2250)	Forward	GGCATGCCATGGGAATCCCATATGATGCGTATCGCATACGCG
	Reverse	CATGCCATGGCATGCCGGATCCCGAACCAACGTTCGGCGC
SsoPCNA1	Forward	GGCATGCCATGGGAATCCCATATGATGTTTAAAATTGTGTATCCGAACGCC
	Reverse	CCATGGCATGCCGGATCCCGACAGGCGCGGCGC
SsoPCNA2	Forward	GGCATGCCATGGGAATCCCATATGATGATGAAAGCCAAAGTGATCG
	Reverse	CCATGGCATGCCGGATCCCGAATCCGCGCGCGG
SsoPCNA3	Forward	GGCATGCCATGGGAATCCCATATGATGATCTACCTGAAATCTTTCGAAC
	Reverse	CCATGGCATGCCGGATCCCGACACTTTCGGCGCCAG

Supplementary Table 6. The Förster resonance energy transfer (**FERT) signals of equimolar protein mixtures.**

PCNA subunit1	PCNA subunit2	PCNA subunit3	FRET signal (I_{528}/I_{477})
SsoPCNA1-CyPet	YPet-SsoPCNA2	-	8.9
YPet-SsoPCNA1	SsoPCNA2-CyPet	-	0.95
-	SsoPCNA2-CyPet	YPet-SsoPCNA3	0.69
SsoPCNA1	SsoPCNA2-CyPet	YPet-SsoPCNA3	6.7
MsePCNA1-CyPet	YPet-MsePCNA2	-	6.6
YPet-MsePCNA1	MsePCNA2-CyPet	-	1.1
-	MsePCNA2-CyPet	YPet-MsePCNA3	1.4
MsePCNA1	MsePCNA2-CyPet	YPet-MsePCNA3	6.4
MsePCNA1-CyPet	YPet-SsoPCNA2	-	7.4
YPet-MsePCNA1	SsoPCNA2-CyPet	-	5.0
MsePCNA1	SsoPCNA2-CyPet	YPet-SsoPCNA3	5.2
SsoPCNA1-CyPet	YPet-MsePCNA2	-	7.1
YPet-SsoPCNA1	MsePCNA2-CyPet	-	0.74
SsoPCNA1	MsePCNA2-CyPet	YPet-SsoPCNA3	5.4
MsePCNA1	MsePCNA2-CyPet	YPet-SsoPCNA3	4.8
MsePCNA1	SsoPCNA2-CyPet	YPet-MsePCNA3	2.8
SsoPCNA1	MsePCNA2-CyPet	YPet-MsePCNA3	3.9
SsoPCNA1	SsoPCNA2-CyPet	YPet-MsePCNA3	1.2

a

```
Msed_0051     1 MFRAIYGSSRDFYYIVSSISKISDELTLNFTEEGIGSKYLTDDKVMMGVV 50
SsoPCNA1      1 MFKIVYPNAKDFFSFINSITNVTDSIILNFTEDGIFSRHLTEDKVLMAIM 50

Msed_0051    51 EIGKDALEEYSIEKPISVKLNLGELKKILSKMKGR-SSVEITETNEGIRI 99
SsoPCNA1     51 RIPKDVLSEYSIDSPTSVKLDVSSVKKILSKASSKKATIELTETDSGLKI 100
                                         α-2

Msed_0051   100 SMKDEKTGTRSSLSIKAEKGEPQILKEPSVAHSVTMGIGGDILSILVDES 149
SsoPCNA1    101 IIRDEKSGAKSTIYIKAEKGQVEQLTEPKVNLAVNFTTDESVLNVIAADV 150
                        β-9                                  α-3

Msed_0051   150 MQVGEEVEIKAEDDHVSFEVEEAGKKYSAVLKNGKPLTKLEIEKQGSSRY 199
SsoPCNA1    151 TLVGEEMRISTEEDKIKIEAGEEGKRYVAFLMKDKPLKELSIDTSASSSY 200
                                          β-13

Msed_0051   200 SLAILEKVSSALS-FSKEIEIGFGAGIPMKLTAPLEKGAGIRFWVAPRL 247
SsoPCNA1    201 SAEMFKDAVKGLRGFSAPTMVSFGENLPMKIDVEAVSGGHMIFWIAPRL 249
```

b

```
Msed_1792     1 -MKFKVIDANSMATIFRTIGEFMPEITLIGTKEGVRLSGVDPARVALIDI 49
SsoPCNA2      1 MMKAKVIDAVSFSYILRTVGDFLSEANFIVTKEGIRVSGIDPSRVVFLDI 50

Msed_1792    50 FIPQAYFHEYES-AEKETVTVKLEEIIASLKNVKKNDSLTFQSGEDRLMI 98
SsoPCNA2     51 FLPSSYFEGFEVSQEKEIIGFKLEDVNDILKRVLKDDTLILSSNESKLTL 100
                                    α-2

Msed_1792    99 TLDGDFERTFYLPILMGEEPSLPSIKLEFAFKAKMLTSTFSNVMQILGDL 148
SsoPCNA2    101 TFDGEFTRSFELPLIQVESTQPPSVNLEFPFKAQLLTITFADIIDELSDL 150
                     β-9                                α-3

Msed_1792   149 GDALTLSAEGGKLTFMVEGDVGSSKVELSEESGTLLEATGADAKGTYGMD 198
SsoPCNA2    151 GEVLNIHSKENKLYFEVIGDLSTAKVELSTDNGTLLEASGADVSSSYGME 200
                                      β-13

Msed_1792   199 YLVKTAKMRNSSDIVELMFGSQLPIKLRFELPQEGYGDFYIAPRAE 244
SsoPCNA2    201 YVANTTKMRRASDSMELYFGSQIPLKLRFKLPQEGYGDFYIAPRAD 246
```

c

```
Msed_2250     1 ---------------MRIAYANAMDFKTVIEALSKLIDEVTFTFTSSGLD 35
SsoPCNA3      1 MIYLKSFERNIRLINMKVVYDDVRVLKDIIQALARLVDEAVLKFKQDSVE 50

Msed_2250    36 VVAVDRAHISLIKLHFPKEAFEEFDVEDQFRFGFNTQYMLKVMASAKRKE 85
SsoPCNA3     51 LVALDRAHISLISVNLPREMFKEYDVNDEFKFGFNTQYLMKILKVAKRKE 100
                                                      α-2

Msed_2250    86 KIEMESREESEIVIRMLGEPPREFTIRNIEVPIQELPELKLDFDVKAKIT 135
SsoPCNA3    101 AIEIASESPDSVIINIIGSTNREFNVRNLEVSEQEIPEINLQFDISATIS 150
                                       β-9

Msed_2250   136 SGGFKKAVSEIATVSDSVEIDATEMEIKLRSKESTEIEVEFSKEMGGLQE 185
SsoPCNA3    151 SDGFKSAISEVSTVTDNVVVEGHEDRILIKAEGESEVEVEFSKDTGGLQD 200
                     α-3                                β-13

Msed_2250   186 IEVKKPSVSSYPSDYLEDVLVLTRLSGFLNLLFSEQKPLQLEFNMDNGGS 235
SsoPCNA3    201 LEFSKESKNSYSAEYLDDVLSLTKLSDYVKISFGNQKPLQLFFNMEGGGK 250

Msed_2250   236 VVYLLAPNVG 245
SsoPCNA3    251 VTYLLAPKV- 259
```

Figure S1. Sequence alignments between the *M. sedula* PCNA homologues and the *S. solfataricus* PCNAs. (**a**) Msed_0051 and SsoPCNA1, (**b**) Msed_1792 and SsoPCNA2, and (**c**) Msed_2250 and SsoPCNA3.

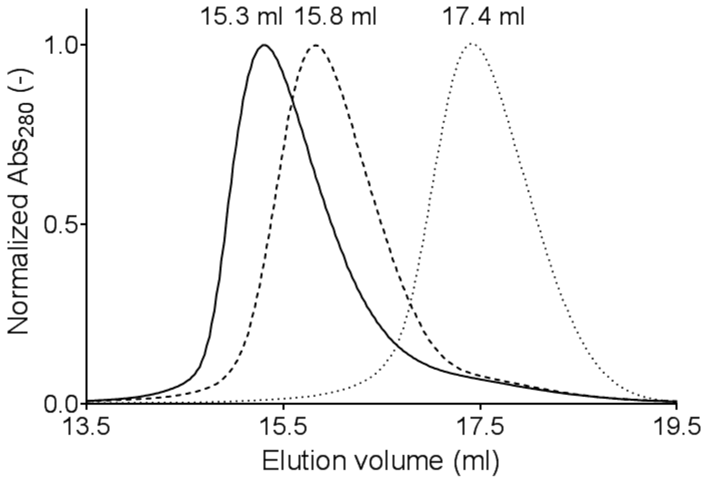

Figure S2. Elution profiles of the SsoPCNA1:SsoPCNA2:SsoPCNA3 heterotrimer (solid line), the SsoPCNA1:SsoPCNA2 heterodimer (broken line) and the SsoPCNA3 monomer (dotted line) from a Superdex 200 10/300 GL column. Protein mixtures containing 50 μM SsoPCNA1, 50 μM SsoPCNA2 and 50 μM SsoPCNA3, or 50 μM SsoPCNA1 and 50 μM SsoPCNA2, or 50 μM SsoPCNA3 in 120 μl of 50 mM potassium phosphate buffer, pH7.4, containing 150 mM KCl were incubated on ice for more than 1 h. Then, 100 μl of the mixtures were subjected to a Superdex 200 10/300 GL column at a flow rate of 1.0 ml/min.

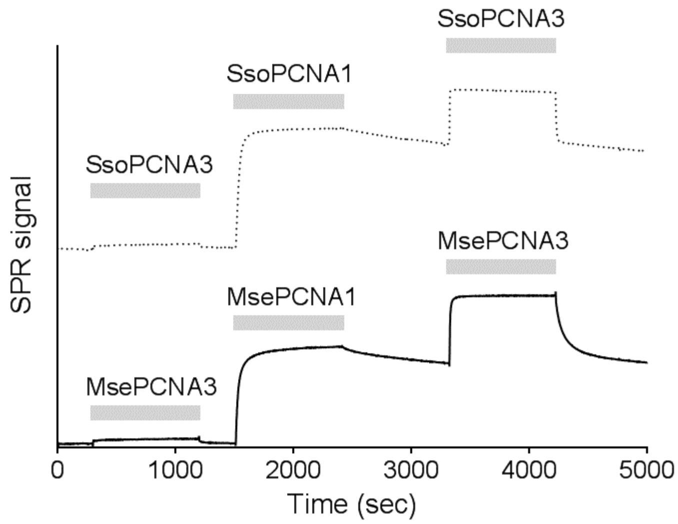

Figure S3. SPR analysis of sequential binding of MsePCNA1 and MsePCNA3 to the immobilised MsePCNA2. MsePCNA3 (400 nM), MsePCNA1 (50 nM) and MsePCNA3 (400 nM) were sequentially injected on a sensor chip immobilising MsePCNA2 (solid line). SsoPCNA3 (400 nM), SsoPCNA1 (50 nM) and SsoPCNA3 (400 nM) were sequentially injected on a sensor chip immobilising SsoPCNA2 (dotted line). Grey lines indicate time periods of protein injection.

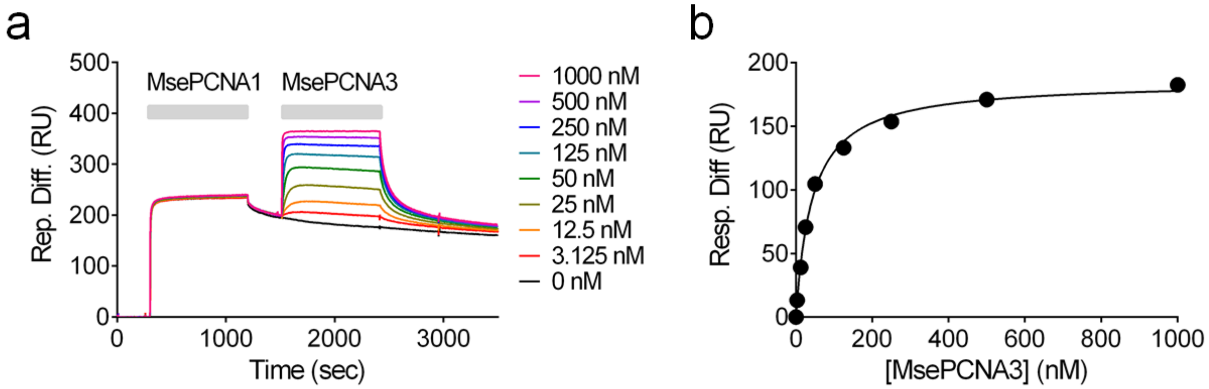

Figure S4. SPR analysis of binding of MsePCNA3 to the MsePCNA1:MsePCNA2 complex. (**a**) Sensorgram of injections of MsePCNA3 (0, 3.125, 12.5, 25, 50, 125, 250, 500, 1000 nM) on a sensor chip coupled to MsePCNA1 through the immobilised MsePCNA2. (**b**) Relationship between the SPR signal change at equilibrium and the MsePCNA3 concentration.

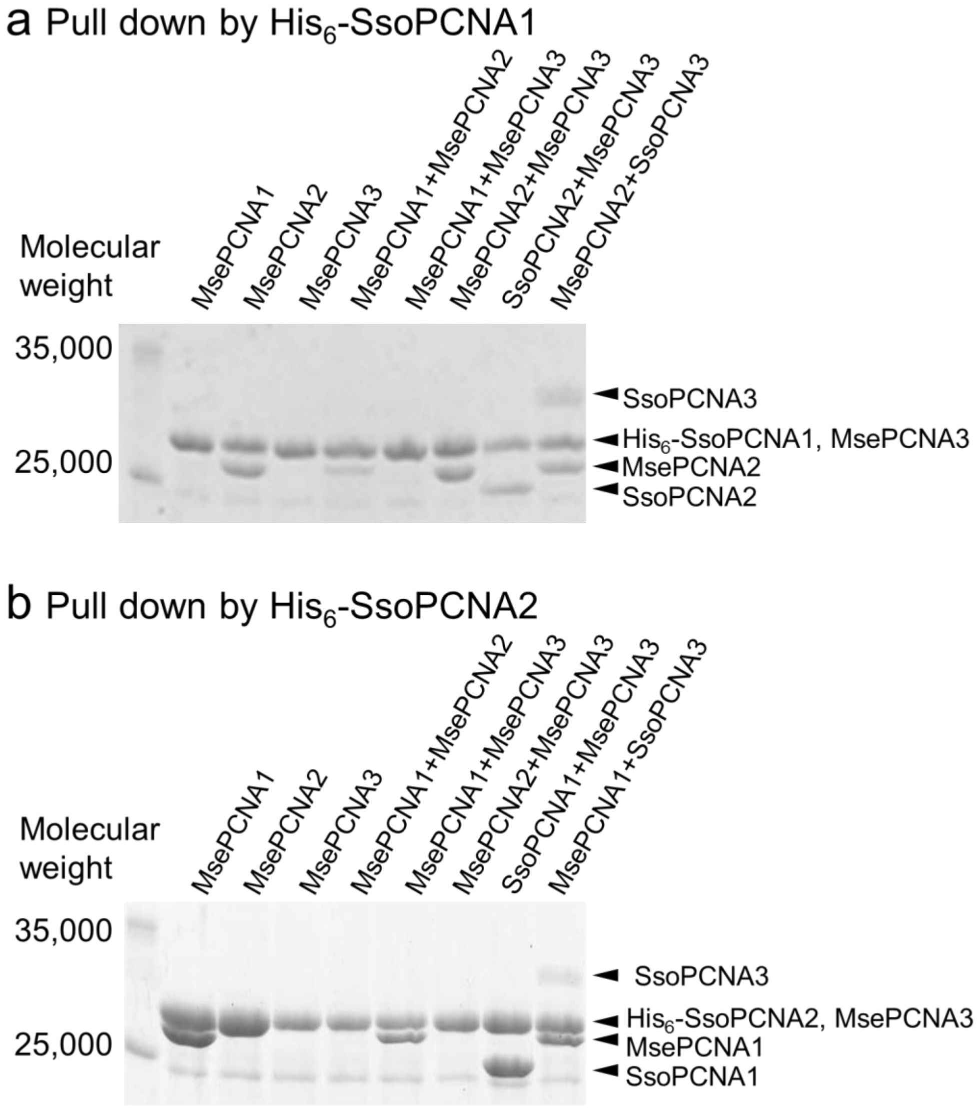

Figure S5. Interactions among the *M. sedula* PCNA homologues and the *S. solfataricus* PCNAs. *M. sedula* PCNA homologues were pulled down by (**a**) His_6-tagged SsoPCNA1 and (**b**) His_6-tagged SsoPCNA2, using Co^{2+}-immobilised resin. Proteins bound to the resin were eluted with 500 mM imidazole and analysed by SDS-PAGE. MsePCNA3 was not separated from His_6-SsoPCNA1 and His6-SsoPCNA2 by SDS-PAGE.

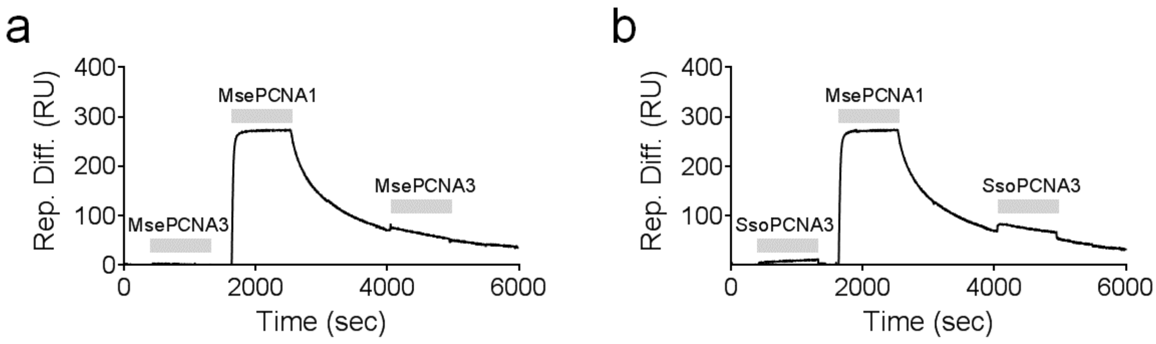

Figure S6. Sensorgrams of sequential injections on the sensor chip immobilising SsoPCNA2. (**a**) MsePCNA3 (400 nM), MsePCNA1 (40 nM) and MsePCNA3 (400 nM), and (**b**) SsoPCNA3 (400 nM), MsePCNA1 (40 nM) and SsoPCNA3 (400 nM) were sequentially injected on the sensor chip. Grey lines indicate time periods of protein injection.

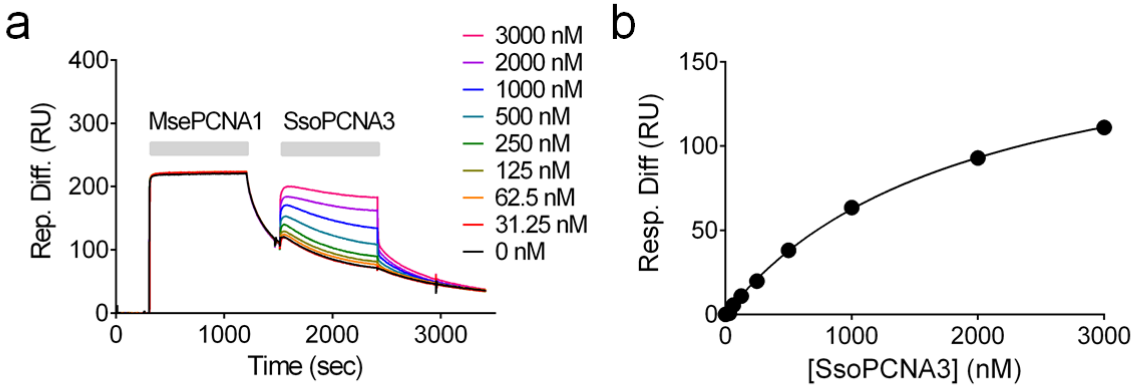

Figure S7. SPR analysis of binding of SsoPCNA3 to the MsePCNA1:MsePCNA2 complex. Sensorgrams of sequential injections of MsePCNA1 (1000 nM) and SsoPCNA3 (0, 31.25, 62.5, 125, 250, 500, 1000, 2000, 3000 nM) on a SsoPCNA2-immobilised sensor chip. (**b**) Relationship between the SPR signal change at equilibrium and the SsoPCNA3 concentration.

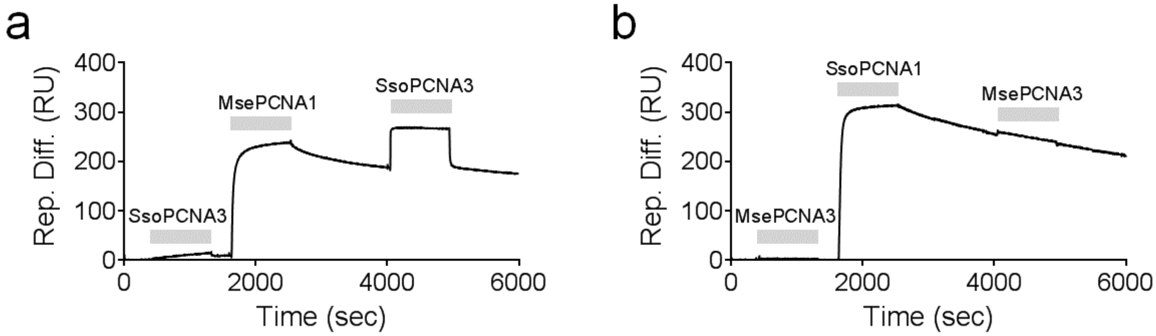

Figure S8. Sensorgrams of sequential injections on the sensor chip immobilising (a) MsePCNA2 and (b) SsoPCNA2. (**a**) SsoPCNA3 (400 nM), MsePCNA1 (50 nM) and SsoPCNA3 (400 nM), and (**b**) MsePCNA3 (400 nM), SsoPCNA1 (50 nM) and MsePCNA3 (400 nM) were sequentially injected over the sensor chip. Grey lines indicate time periods of protein injection.

Protein sequences

His$_6$-tagged MsePCNA1

MGSSHHHHHHSSGLVPRGSHSENLYFQGHMFRAIYGSSRDFYYIVSSISKISDETTTN
FTEEGIGSKYTTDDKVMMGVVEIGKDATEEYSIEKPISVKTNTGETKKITSKMKGRSSV
EITETNEGIRISMKDEKTGTRSSTSIKAEKGEPQITKEPSVAHSVTMGIGGDITSITVDES
MQVGEEVEIKAEDDHVSFEVEEAGKKYSAVTKNGKPTTKTEIEKQGSSRYSTAITEKVS
SATSFSKEIEIGFGAGIPMKTTAPTEKGAGIRFWVAPRT

MsePCNA1

HMFRAIYGSSRDFYYIVSSISKISDETTTNFTEEGIGSKYTTDDKVMMGVVEIGKDATEE
YSIEKPISVKTNTGETKKITSKMKGRSSVEITETNEGIRISMKDEKTGTRSSTSIKAEKGE
PQITKEPSVAHSVTMGIGGDITSITVDESMQVGEEVEIKAEDDHVSFEVEEAGKKYSAV
TKNGKPTTKTEIEKQGSSRYSTAITEKVSSATSFSKEIEIGFGAGIPMKTTAPTEKGAGIR
FWVAPRT

His$_6$-tagged MsePCNA2

MGSSHHHHHHSSGLVPRGSHSENLYFQGHMKFKVIDANSMATIFRTIGEFMPEITTIGT
KEGVRTSGVDPARVATIDIFIPQAYFHEYESAEKETVTVKTEEIIASTKNVKKNDSTTFQ
SGEDRTMITTDGDFERTFYTPITMGEEPSTPSIKTEFAFKAKMTTSTFSNVMQITGDTG
DATTTSAEGGKTTFMVEGDVGSSKVETSEESGTTTEATGADAKGTYGMDYTVKTAKM
RNSSDIVETMFGSQTPIKTRFETPQEGYGDFYIAPRAE

MsePCNA2

HMKFKVIDANSMATIFRTIGEFMPEITTIGTKEGVRTSGVDPARVATIDIFIPQAYFHEYE
SAEKETVTVKTEEIIASTKNVKKNDSTTFQSGEDRTMITTDGDFERTFYTPITMGEEPST
PSIKTEFAFKAKMTTSTFSNVMQITGDTGDATTTSAEGGKTTFMVEGDVGSSKVETSE
ESGTTTEATGADAKGTYGMDYTVKTAKMRNSSDIVETMFGSQTPIKTRFETPQEGYG
DFYIAPRAE

His$_6$-tagged MsePCNA3

MGSSHHHHHHSSGLVPRGSHSENLYFQGHMRIAYANAMDFKTVIEATSKTIDEVTFTF
TSSGTDVVAVDRAHISTIKTHFPKEAFEEFDVEDQFRFGFNTQYMTKVMASAKRKEKIE
MESREESEIVIRMTGEPPREFTIRNIEVPIQETPETKTDFDVKAKITSGGFKKAVSEIATV
SDSVEIDATEMEIKTRSKESTEIEVEFSKEMGGTQEIEVKKPSVSSYPSDYTEDVTVTTR
TSGFTNTTFSEQKPTQTEFNMDNGGSVVYTTAPNVG

MsePCNA3

GSHSENLYFQGHMRIAYANAMDFKTVIEATSKTIDEVTFTFTSSGTDVVAVDRAHISTIK
THFPKEAFEEFDVEDQFRFGFNTQYMTKVMASAKRKEKIEMESREESEIVIRMTGEPP
REFTIRNIEVPIQETPETKTDFDVKAKITSGGFKKAVSEIATVSDSVEIDATEMEIKTRSKE
STEIEVEFSKEMGGTQEIEVKKPSVSSYPSDYTEDVTVTTRTSGFTNTTFSEQKPTQTE
FNMDNGGSVVYTTAPNVG

His$_6$-tagged SsoPCNA1

MGSSHHHHHHSSGLVPRGSHSENLYFQGHMFKIVYPNAKDFFSFINSITNVTDSIILNF
TEDGIFSRHLTEDKVLMAIMRIPKDVLSEYSIDSPTSVKLDVSSVKKILSKASSKKATIELT

ETDSGLKIIIRDEKSGAKSTIYIKAEKGQVEQLTEPKVNLAVNFTTDESVLNVIAADVTLV
GEEMRISTEEDKIKIEAGEEGKRYVAFLMKDKPLKELSIDTSASSSYSAEMFKDAVKGL
RGFSAPTMVSFGENLPMKIDVEAVSGGHMIFWIAPRL

SsoPCNA1
HMFKIVYPNAKDFFSFINSITNVTDSIILNFTEDGIFSRHLTEDKVLMAIMRIPKDVLSEYSI
DSPTSVKLDVSSVKKILSKASSKKATIELTETDSGLKIIIRDEKSGAKSTIYIKAEKGQVEQ
LTEPKVNLAVNFTTDESVLNVIAADVTLVGEEMRISTEEDKIKIEAGEEGKRYVAFLMKD
KPLKELSIDTSASSSYSAEMFKDAVKGLRGFSAPTMVSFGENLPMKIDVEAVSGGHMIF
WIAPRL

His_6-tagged SsoPCNA2
MGSSHHHHHHSSGLVPRGSHSENLYFQGHMMKAKVIDAVSFSYILRTVGDFLSEANFI
VTKEGIRVSGIDPSRVVFLDIFLPSSYFEGFEVSQEKEIIGFKLEDVNDILKRVLKDDTLIL
SSNESKLTLTFDGEFTRSFELPLIQVESTQPPSVNLEFPFKAQLLTITFADIIDELSDLGE
VLNIHSKENKLYFEVIGDLSTAKVELSTDNGTLLEASGADVSSSYGMEYVANTTKMRRA
SDSMELYFGSQIPLKLRFKLPQEGYGDFYIAPRAD

SsoPCNA2
HMMKAKVIDAVSFSYILRTVGDFLSEANFIVTKEGIRVSGIDPSRVVFLDIFLPSSYFEGF
EVSQEKEIIGFKLEDVNDILKRVLKDDTLILSSNESKLTLTFDGEFTRSFELPLIQVESTQP
PSVNLEFPFKAQLLTITFADIIDELSDLGEVLNIHSKENKLYFEVIGDLSTAKVELSTDNGT
LLEASGADVSSSYGMEYVANTTKMRRASDSMELYFGSQIPLKLRFKLPQEGYGDFYIA
PRAD

His_6-tagged SsoPCNA3
MGSSHHHHHHSSGLVPRGSHSENLYFQGHMIYLKSFERNIRLINMKVVYDDVRVLKDII
QALARLVDEAVLKFKQDSVELVALDRAHISLISVNLPREMFKEYDVNDEFKFGFNTQYL
MKILKVAKRKEAIEIASESPDSVIINIIGSTNREFNVRNLEVSEQEIPEINLQFDISATISSD
GFKSAISEVSTVTDNVVVEGHEDRILIKAEGESEVEVEFSKDTGGLQDLEFSKESKNSY
SAEYLDDVLSLTKLSDYVKISFGNQKPLQLFFNMEGGGKVTYLLAPKV

SsoPCNA3
HMIYLKSFERNIRLINMKVVYDDVRVLKDIIQALARLVDEAVLKFKQDSVELVALDRAHIS
LISVNLPREMFKEYDVNDEFKFGFNTQYLMKILKVAKRKEAIEIASESPDSVIINIIGSTNR
EFNVRNLEVSEQEIPEINLQFDISATISSDGFKSAISEVSTVTDNVVVEGHEDRILIKAEG
ESEVEVEFSKDTGGLQDLEFSKESKNSYSAEYLDDVLSLTKLSDYVKISFGNQKPLQLF
FNMEGGGKVTYLLAPKV

Ypet-SsoPCNA1
MGSSHHHHHHSSGLVPRGSHMMVSKGEELFTGVVPILVELDGDVNGHKFSVSGEGE
GDATYGKLTLKLLCTTGKLPVPWPTLVTTLGYGLQCFARYPDHMKQHDFFKSAMPEG
YVQERTIFFKDDGNYKTRAEVKFEGDTLVNRIELKGIDFKEDGNILGHKLEYNYNSHNV
YITADKQKNGIKANFKIRHNIEDGGVQLADHYQQNTPIGDGPVLLPDNHYLSYQSALFK
DPNEKRDHMVLLEFLTAAGITEGMNELYKKGSGMPWESHMFKIVYPNAKDFFSFINSIT
NVTDSIILNFTEDGIFSRHLTEDKVLMAIMRIPKDVLSEYSIDSPTSVKLDVSSVKKILSKA

SSKKATIELTETDSGLKIIIRDEKSGAKSTIYIKAEKGQVEQLTEPKVNLAVNFTTDESVLN
VIAADVTLVGEEMRISTEEDKIKIEAGEEGKRYVAFLMKDKPLKELSIDTSASSSYSAEM
FKDAVKGLRGFSAPTMVSFGENLPMKIDVEAVSGGHMIFWIAPRL

Ypet-SsoPCNA2
MGSSHHHHHHSSGLVPRGSHMMVSKGEELFTGVVPILVELDGDVNGHKFSVSGEGE
GDATYGKLTLKLLCTTGKLPVPWPTLVTTLGYGLQCFARYPDHMKQHDFFKSAMPEG
YVQERTIFFKDDGNYKTRAEVKFEGDTLVNRIELKGIDFKEDGNILGHKLEYNYNSHNV
YITADKQKNGIKANFKIRHNIEDGGVQLADHYQQNTPIGDGPVLLPDNHYLSYQSALFK
DPNEKRDHMVLLEFLTAAGITEGMNELYKKGSGMPWESHMMKAKVIDAVSFSYILRTV
GDFLSEANFIVTKEGIRVSGIDPSRVVFLDIFLPSSYFEGFEVSQEKEIIGFKLEDVNDILK
RVLKDDTLILSSNESKLTLTFDGEFTRSFELPLIQVESTQPPSVNLEFPFKAQLLTITFADI
IDELSDLGEVLNIHSKENKLYFEVIGDLSTAKVELSTDNGTLLEASGADVSSSYGMEYVA
NTTKMRRASDSMELYFGSQIPLKLRFKLPQEGYGDFYIAPRAD

Ypet-MsePCNA1
MGSSHHHHHHSSGLVPRGSHMMVSKGEELFTGVVPILVELDGDVNGHKFSVSGEGE
GDATYGKLTLKLLCTTGKLPVPWPTLVTTLGYGLQCFARYPDHMKQHDFFKSAMPEG
YVQERTIFFKDDGNYKTRAEVKFEGDTLVNRIELKGIDFKEDGNILGHKLEYNYNSHNV
YITADKQKNGIKANFKIRHNIEDGGVQLADHYQQNTPIGDGPVLLPDNHYLSYQSALFK
DPNEKRDHMVLLEFLTAAGITEGMNELYKKGSGMPWESHMFRAIYGSSRDFYYIVSSI
SKISDETTTNFTEEGIGSKYTTDDKVMMGVVEIGKDATEEYSIEKPISVKTNTGETKKITS
KMKGRSSVEITETNEGIRISMKDEKTGTRSSTSIKAEKGEPQITKEPSVAHSVTMGIGG
DITSITVDESMQVGEEVEIKAEDDHVSFEVEEAGKKYSAVTKNGKPTTKTEIEKQGSSR
YSTAITEKVSSATSFSKEIEIGFGAGIPMKTTAPTEKGAGIRFWVAPRT

Ypet-MsePCNA2
MGSSHHHHHHSSGLVPRGSHMMVSKGEELFTGVVPILVELDGDVNGHKFSVSGEGE
GDATYGKLTLKLLCTTGKLPVPWPTLVTTLGYGLQCFARYPDHMKQHDFFKSAMPEG
YVQERTIFFKDDGNYKTRAEVKFEGDTLVNRIELKGIDFKEDGNILGHKLEYNYNSHNV
YITADKQKNGIKANFKIRHNIEDGGVQLADHYQQNTPIGDGPVLLPDNHYLSYQSALFK
DPNEKRDHMVLLEFLTAAGITEGMNELYKKGSGMPWESHMKFKVIDANSMATIFRTIG
EFMPEITTIGTKEGVRTSGVDPARVATIDIFIPQAYFHEYESAEKETVTVKTEEIIASTKNV
KKNDSTTFQSGEDRTMITTDGDFERTFYTPITMGEEPSTPSIKTEFAFKAKMTTSTFSN
VMQITGDTGDATTTSAEGGKTTFMVEGDVGSSKVETSEESGTTTEATGADAKGTYGM
DYTVKTAKMRNSSDIVETMFGSQTPIKTRFETPQEGYGDFYIAPRAE

SsoPCNA1-CyPet
MGSSHHHHHHSSGLVPRGSHMFKIVYPNAKDFFSFINSITNVTDSIILNFTEDGIFSRHL
TEDKVLMAIMRIPKDVLSEYSIDSPTSVKLDVSSVKKILSKASSKKATIELTETDSGLKIIIR
DEKSGAKSTIYIKAEKGQVEQLTEPKVNLAVNFTTDESVLNVIAADVTLVGEEMRISTEE
DKIKIEAGEEGKRYVAFLMKDKPLKELSIDTSASSSYSAEMFKDAVKGLRGFSAPTMVS
FGENLPMKIDVEAVSGGHMIFWIAPRLKGSGSGMPWMVSKGEELFGGIVPILVELEGD
VNGHKFSVSGEGEGDATYGKLTLKFICTTGKLPVPWPTLVTTLTWGVQCFSRYPDHM
KQHDFFKSVMPEGYVQERTIFFKDDGNYKTRAEVKFEGDTLVNRIELKGIDFKEDGNIL

GHKLEYNYISHNVYITADKQKNGIKANFKARHNITDGSVQLADHYQQNTPIGDGPVILP
DNHYLSTQSALSKDPNEKRDHMVLLEFVTAAGITLGMDELYKLEDP

SsoPCNA2-CyPet
MGSSHHHHHHSSGLVPRGSHMMKAKVIDAVSFSYILRTVGDFLSEANFIVTKEGIRVS
GIDPSRVVFLDIFLPSSYFEGFEVSQEKEIIGFKLEDVNDILKRVLKDDTLILSSNESKLTL
TFDGEFTRSFELPLIQVESTQPPSVNLEFPFKAQLLTITFADIIDELSDLGEVLNIHSKENK
LYFEVIGDLSTAKVELSTDNGTLLEASGADVSSSYGMEYVANTTKMRRASDSMELYFG
SQIPLKLRFKLPQEGYGDFYIAPRADKGSGSGMPWMVSKGEELFGGIVPILVELEGDV
NGHKFSVSGEGEGDATYGKLTLKFICTTGKLPVPWPTLVTTLTWGVQCFSRYPDHMK
QHDFFKSVMPEGYVQERTIFFKDDGNYKTRAEVKFEGDTLVNRIELKGIDFKEDGNILG
HKLEYNYISHNVYITADKQKNGIKANFKARHNITDGSVQLADHYQQNTPIGDGPVILPDN
HYLSTQSALSKDPNEKRDHMVLLEFVTAAGITLGMDELYKLEDP

SsoPCNA3-CyPet
MGSSHHHHHHSSGLVPRGSHMIYLKSFERNIRLINMKVVYDDVRVLKDIIQALARLVDE
AVLKFKQDSVELVALDRAHISLISVNLPREMFKEYDVNDEFKFGFNTQYLMKILKVAKR
KEAIEIASESPDSVIINIIGSTNREFNVRNLEVSEQEIPEINLQFDISATISSDGFKSAISEVS
TVTDNVVVEGHEDRILIKAEGESEVEVEFSKDTGGLQDLEFSKESKNSYSAEYLDDVLS
LTKLSDYVKISFGNQKPLQLFFNMEGGGKVTYLLAPKKGSGSGMPWMVSKGEELFGG
IVPILVELEGDVNGHKFSVSGEGEGDATYGKLTLKFICTTGKLPVPWPTLVTTLTWGVQ
CFSRYPDHMKQHDFFKSVMPEGYVQERTIFFKDDGNYKTRAEVKFEGDTLVNRIELKG
IDFKEDGNILGHKLEYNYISHNVYITADKQKNGIKANFKARHNITDGSVQLADHYQQNTP
IGDGPVILPDNHYLSTQSALSKDPNEKRDHMVLLEFVTAAGITLGMDELYKLEDP

MsePCNA1-CyPet
MGSSHHHHHHSSGLVPRGSHMFRAIYGSSRDFYYIVSSISKISDETTTNFTEEGIGSKY
TTDDKVMMGVVEIGKDATEEYSIEKPISVKTNTGETKKITSKMKGRSSVEITETNEGIRIS
MKDEKTGTRSSTSIKAEKGEPQITKEPSVAHSVTMGIGGDITSITVDESMQVGEEVEIKA
EDDHVSFEVEEAGKKYSAVTKNGKPTTKTEIEKQGSSRYSTAITEKVSSATSFSKEIEIG
FGAGIPMKTTAPTEKGAGIRFWVAPRTKGSGSGMPWMVSKGEELFGGIVPILVELEGD
VNGHKFSVSGEGEGDATYGKLTLKFICTTGKLPVPWPTLVTTLTWGVQCFSRYPDHM
KQHDFFKSVMPEGYVQERTIFFKDDGNYKTRAEVKFEGDTLVNRIELKGIDFKEDGNIL
GHKLEYNYISHNVYITADKQKNGIKANFKARHNITDGSVQLADHYQQNTPIGDGPVILP
DNHYLSTQSALSKDPNEKRDHMVLLEFVTAAGITLGMDELYKLEDP

MsePCNA2-CyPet
MGSSHHHHHHSSGLVPRGSHMKFKVIDANSMATIFRTIGEFMPEITTIGTKEGVRTSGV
DPARVATIDIFIPQAYFHEYESAEKETVTVKTEEIIASTKNVKKNDSTTFQSGEDRTMITT
DGDFERTFYTPITMGEEPSTPSIKTEFAFKAKMTTSTFSNVMQITGDTGDATTTSAEGG
KTTFMVEGDVGSSKVETSEESGTTTEATGADAKGTYGMDYTVKTAKMRNSSDIVETM
FGSQTPIKTRFETPQEGYGDFYIAPRAEKGSGSGMPWMVSKGEELFGGIVPILVELEG
DVNGHKFSVSGEGEGDATYGKLTLKFICTTGKLPVPWPTLVTTLTWGVQCFSRYPDH
MKQHDFFKSVMPEGYVQERTIFFKDDGNYKTRAEVKFEGDTLVNRIELKGIDFKEDGNI

LGHKLEYNYISHNVYITADKQKNGIKANFKARHNITDGSVQLADHYQQNTPIGDGPVILP
DNHYLSTQSALSKDPNEKRDHMVLLEFVTAAGITLGMDELYKLEDP

MsePCNA3-CyPet
MGSSHHHHHHSSGLVPRGSHMRIAYANAMDFKTVIEATSKTIDEVTFTFTSSGTDVVA
VDRAHISTIKTHFPKEAFEEFDVEDQFRFGFNTQYMTKVMASAKRKEKIEMESREESEI
VIRMTGEPPREFTIRNIEVPIQETPETKTDFDVKAKITSGGFKKAVSEIATVSDSVEIDAT
EMEIKTRSKESTEIEVEFSKEMGGTQEIEVKKPSVSSYPSDYTEDVTVTTRTSGFTNTT
FSEQKPTQTEFNMDNGGSVVYTTAPNVGKGSGSGMPWMVSKGEELFGGIVPILVELE
GDVNGHKFSVSGEGEGDATYGKLTLKFICTTGKLPVPWPTLVTTLTWGVQCFSRYPD
HMKQHDFFKSVMPEGYVQERTIFFKDDGNYKTRAEVKFEGDTLVNRIELKGIDFKEDG
NILGHKLEYNYISHNVYITADKQKNGIKANFKARHNITDGSVQLADHYQQNTPIGDGPVI
LPDNHYLSTQSALSKDPNEKRDHMVLLEFVTAAGITLGMDELYKLEDP

MseLig1
MGSSHHHHHHSSGLVPRGSHMKFKLIAEYFDRLEKISSRIQLTSLLSDLFKNTEREVID
KVVYLIQGRLWPDFTGMPEIGMGEKFLIKAIAMAYGNKEEEVEKLYKNIGDLGEVAYSL
RSKVKGVSILSFVGGNQEAGELDVMEVYNELVKIATSTGEGSRDIKIRIFAGLIKKATPIE
AKYLVRFVEGRLRLGIGDATVLDALAITFGGSADYRPIVERAYNLRADLGDIARVIATEGI
EKLKNISPTPGIPIRPMLAERLPDPEEIMEKMNGKALVDYKYDGERAQIHRKGDKVTIFS
RRMENITDQYIDVTEYVKQFVKGDNFIVEGEIVPVDPESGEMRPFQELMHRRRKNNIA
EAIKEYPVNLFLFDLMFFEGEDYTTKPLPERRAKLEEILASNDKVHIASHIIADRVDKLRE
YFYQAISEGAEGVMVKSIGPDSIYQAGSRGWLWIKLKRDYQSEMADTVDLVVVGAFYG
KGKRGGKFSSLLMAAYNPEKDVFETVCKVASGFSDQELDEMQKKINELKREQKHPRV
VSDMIPDVWVSPTLVAEVIGAEITISPLHTCCRGEKGGLSIRFPRFIRWRDDKSPEDATT
NQEIMEMYSKQLKKIEEKPVDENI

*YPet, CyPet and peptide linkers connecting fluorescent proteins and PCNA proteins are highlighted in yellow, cyan and green, respectively.

Supplementary references

S1. Haga, T., Hirakawa, H. & Nagamune, T. Fine tuning of spatial arrangement of enzymes in a PCNA-mediated multienzyme complex using a rigid poly-L-proline linker. *PLoS One* **8,** e75114 (2013).

S2. Hirakawa, H., Kakitani, A. & Nagamune, T. Introduction of selective intersubunit disulfide bonds into self-assembly protein scaffold to enhance an artificial multienzyme complex's activity. *Biotechnol. Bioeng.* **110,** 1858–1864 (2013).

S3. Kapust, R. B. *et al.* Tobacco etch virus protease: mechanism of autolysis and rational design of stable mutants with wild-type catalytic proficiency. *Protein Eng.* **14,** 993–1000 (2001).

S4. Hirakawa, H. & Nagamune, T. Molecular assembly of P450 with ferredoxin and ferredoxin reductase by fusion to PCNA. *Chembiochem* **11,** 1517–1520 (2010).
